# Restriction of Individual Branched‐Chain Amino Acids has Distinct Effects on the Development and Progression of Alzheimer's Disease in 3xTg Mice

**DOI:** 10.1002/advs.202515220

**Published:** 2026-03-12

**Authors:** Reji Babygirija, Cara L. Green, Michelle M. Sonsalla, Izabelle Marie F. Le, Fan Xiao, Sarah Yandell, Mariah F. Calubag, Michaela E. Trautman, Anna Tobon, Ryan Matoska, Chung‐Yang Yeh, Charles I. Opara, Isaac Grunow, Diana Vertein, Sophia Schlorf, Bailey A. Knopf, Michael J. Rigby, David A. Harris, Mark P. Keller, Alan D. Attie, Luigi Puglielli, Cholsoon Jang, Dudley W. Lamming

**Affiliations:** ^1^ Department of Medicine University of Wisconsin‐Madison Madison Wisconsin USA; ^2^ William S. Middleton Memorial Veterans Hospital Madison Wisconsin USA; ^3^ Cellular and Molecular Biology Graduate Program University of Wisconsin‐Madison Madison Wisconsin USA; ^4^ Comparative Biomedical Sciences Graduate Program University of Wisconsin‐Madison Madison Wisconsin USA; ^5^ Department of Biological Chemistry University of California Irvine California USA; ^6^ Nutrition and Metabolism Graduate Program University of Wisconsin‐Madison Madison Wisconsin USA; ^7^ Department of Biochemistry University of Wisconsin‐Madison Madison Wisconsin USA; ^8^ Waisman Center University of Wisconsin‐Madison Madison Wisconsin USA; ^9^ Neuroscience Training Program University of Wisconsin‐Madison Madison Wisconsin USA; ^10^ Wisconsin Surgical Laboratory in Metabolism Department of Surgery University of Wisconsin‐Madison Madison Wisconsin USA; ^11^ Wisconsin Nathan Shock Center of Excellence in the Basic Biology of Aging Madison Wisconsin USA; ^12^ Comprehensive Diabetes Center University of Wisconsin Madison Wisconsin USA; ^13^ Carbone Cancer Center University of Wisconsin Madison Wisconsin USA

**Keywords:** Alzheimer's disease, autophagy, branched chain amino acids, mTORC1

## Abstract

Dietary protein regulates metabolic health and aging, with many benefits of a low protein diet resulting from reduced consumption of the three branched‐chain amino acids (BCAAs), leucine, isoleucine, and valine. Each BCAA has distinct physiological and molecular effects, and while restriction of protein or all three BCAAs improves cognition in mouse models of Alzheimer's disease (AD), the role of each individual BCAA on AD is unknown. Here, we investigate the impact of restricting leucine, isoleucine, or valine on metabolism, AD pathology, molecular signaling, and cognition in male and female 3xTg AD mice. Mice were fed BCAA‐restricted diets for nine months starting at six months of age. Restriction of either isoleucine or valine, but not leucine, improved metabolic health. We observed distinct, BCAA‐specific effects on AD pathology, molecular signaling, and gene expression in both sexes as well as shared molecular responses in males. Restricting any BCAA improved short‐term memory in males, with isoleucine having the strongest effect, while valine restriction led to the greatest cognitive benefits for females. These findings suggest that targeted BCAA restriction, particularly of isoleucine or valine, may form the basis of a novel sex‐specific approach to prevent or delay AD.

## Graphical abstract

Protein restriction (PR) slows Alzheimer's disease (AD) in mice, and other benefits of PR are due to decreased branched‐chain amino acids (BCAAs). We show that restricting any BCAA has benefits, with sex‐ and BCAA‐specific impacts on pathology, molecular signaling, and cognition. These findings highlight dietary composition as critical in the development and progression of AD, and could guide future therapies.

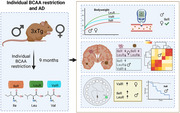

## 1 Introduction

Currently, around 6.9 million Americans age 65 and older are living with Alzheimer's disease (AD)—a number that could double to 13.8 million by 2060 if no significant medical breakthroughs are made to prevent or cure the disease [1]. While progress has been made in identifying effective interventions, including the discovery of monoclonal antibodies that may slow AD progression, these treatments come with significant side effects, high costs, and limited efficacy, making the search for more precise and effective treatments urgent [2, 3].

Dietary interventions can be more affordable and effective than pharmaceuticals for disease management. Specifically, calorie restriction (CR) has been shown to delay or prevent Alzheimer's Disease (AD) progression in various murine models [4, 5, 6, 7]. While a CR diet is difficult for many people to adhere to, diets with altered levels of specific macronutrients that do not restrict calories may be easier to follow [8]. Dietary protein restriction (PR) promotes metabolic health in both humans and mice, and extends lifespan in mice [9, 10, 11, 12, 13, 14]. We recently showed that PR slows progression of AD pathology and preserves cognition in the 3xTg mouse model of AD [15].

The metabolic benefits of PR are driven in part by reduced intake of the branched‐chain amino acids (BCAAs; leucine, isoleucine, and valine), and restriction of the BCAAs is sufficient to recapitulate the effects of PR on healthspan and lifespan in mice [10, 13]. In AD mouse models, BCAA restriction has been shown to improve cognitive function, brain AD pathology, and neurotransmitter levels, suggesting a causal link between BCAAs and AD progression [16]. Impaired BCAA metabolism or excessive dietary BCAAs may drive AD pathogenesis by elevating BCAA levels in the brain and activating the protein kinase mechanistic target of rapamycin complex 1 (mTORC1), a key regulator of many metabolic processes; mTORC1 signaling is upregulated in the brains of both AD patients and AD mouse models [17, 18, 19, 20, 21]. In the 3xTg mouse model of AD, BCAA supplementation worsens AD neuropathology and reduces survival, whereas BCAA restriction improves cognitive deficits [21].

While the BCAAs have often been considered as a group, growing evidence suggests that individual BCAAs have distinctive molecular and physiological roles. For example, we have identified distinct roles for the different BCAAs in the response to PR, showing that restriction of isoleucine is necessary and sufficient for the metabolic benefits of PR, and that isoleucine restriction can extend the lifespan and healthspan of mice [22, 23, 24]. The role of each individual BCAA in the development and progression of AD remains unknown. While all BCAAs can activate mTORC1, they do so to different degrees [25]. Although BCAA catabolism shares many steps, the intermediate and final products of each BCAA are distinct; they are catabolized either to acetyl‐CoA (leucine), propionyl‐CoA (valine), or both (isoleucine), and different tissues catabolize different BCAAs preferentially and at different rates [26]. Furthermore, there is conflicting evidence regarding individual BCAAs in AD pathogenesis in humans. For instance, analysis of cerebrospinal fluid and plasma amino acid profiles revealed a significant reduction in valine levels in AD patients compared to healthy controls [27], while elevated isoleucine levels have been observed in individuals with mild cognitive impairment [28]. Collectively, this suggested to us that it was imperative to investigate the role of individual BCAAs in the development and progression of AD.

In this study, we investigated whether the restriction of individual BCAAs could slow or prevent the progression of AD pathology and cognitive loss in the 3xTg mouse model of AD. This model expresses familial human isoforms of APP (APPSwe), Tau (tauP301L), and Presenilin (PS1M146V), and exhibits Aβ and tau pathology in addition to cognitive deficits [29, 30]. We fed 3xTg mice as well as non‐transgenic (NTg) controls of both sexes either a Control amino acid (AA) defined diet, or diets low in either isoleucine, leucine, or valine starting at 6 months of age (the age at which 3xTg mice begin to develop cognitive deficits and aspects of AD pathology). We assessed the effects of restricting each BCAA on metabolic health, AD neuropathology, cognition, and survival. While the effects of each BCAA vary with sex and the specific assay performed, we generally find that restriction of isoleucine and valine, but not restriction of leucine, promotes metabolic health. Isoleucine and valine showed sex‐specific effects on both AD pathology and molecular signaling. Restricting any of the three BCAAs improved short‐term memory in males, with restriction of isoleucine showing the most pronounced benefits for memory in males; in contrast, restriction of valine showed the greatest benefits for cognition in females. We also found that restricting isoleucine improved the survival of males. Analysis of the brain transcriptome revealed both distinct and shared molecular pathways among all three individual BCAAs in a highly sex‐specific manner. We identified shared and BCAA‐specific pathways associated with improved memory and reduced AD pathology. These findings highlight the distinct impact of each BCAA on metabolic health, AD pathology, and cognition, and their sex‐specific impact, and suggest that restriction of valine in females or isoleucine in males could serve as a novel approach to prevent or delay the progression of AD.

## 2 Results

### 2.1 Restricting Individual BCAAs has Distinct and Sex‐Specific Effects on the Metabolic Health of 3xTg Mice

**FIGURE 1:**
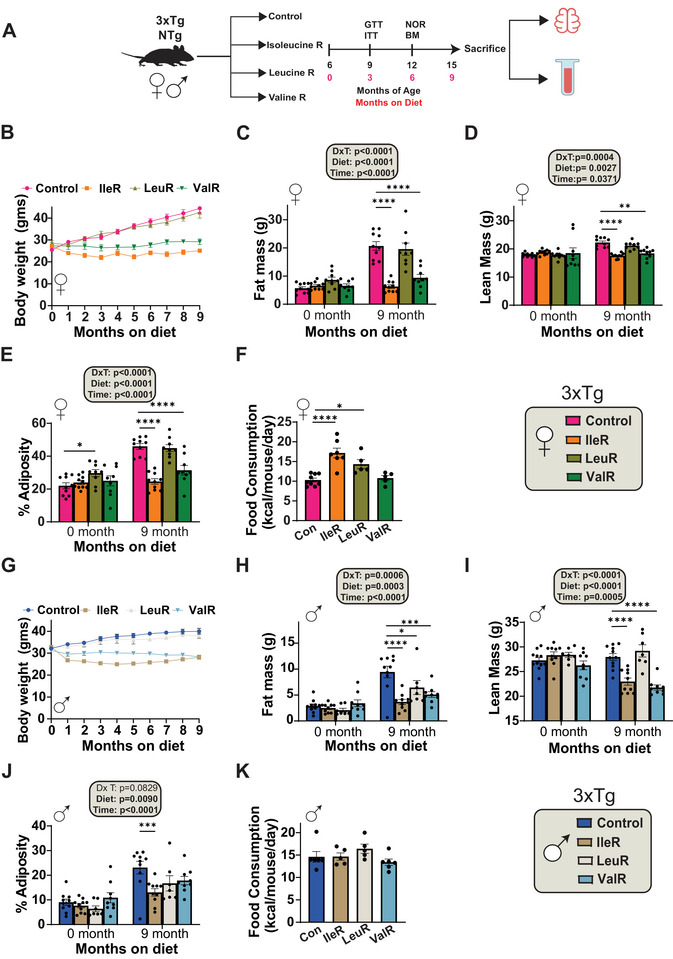
Metabolic health outcomes of 3xTg‐AD mice following individual BCAA restrictions. (A) Experimental design: Six‐month‐old female and male 3xTg‐AD mice were placed on an amino acid‐defined Control (Con) diet or on a diet with a 67% reduction of either isoleucine (IleR), leucine (LeuR), or valine (ValR), and phenotyped over the course of the next 9 months. (B–E) The body weight (B) of female mice was followed over the course of the experiment, fat mass (C) and lean mass (D) were determined at the start and end of the experiment, and the adiposity (E) was calculated. (B–E) *n* = 10 Con, *n *= 10 IleR, *n* = 9 LeuR and *n *= 9 ValR fed 3xTg biologically independent mice. (F) Food consumption of female mice *n* = 9 Con, *n* = 7 IleR, *n* = 5 LeuR, and *n *= 5 ValR fed 3xTg biologically independent mice. (G–J) The body weight (G) of male mice was followed over the course of the experiment, fat mass (H) and lean mass (I) were determined at the start and end of the experiment, and the adiposity (J) was calculated. (G–J) *n *= 11 Con, *n *= 10 IleR, *n *= 7 LeuR and *n *= 9 ValR 3xTg biologically independent mice. (K) Food consumption of male *n *= 7 Con, *n *= 5 IleR, *n *= 5 LeuR, and *n *= 6 ValR 3xTg biologically independent mice. (C–E, H–J) Statistics for the overall effect of diet and time represent the *p* value from a two‐way analysis of variance (ANOVA); *p < 0.05, statistics for the overall effects of diet and time represent the *p* value from a two‐way ANOVA conducted separately for each time point; *p < 0.05, **p < 0.01, ***p < 0.001, **** *p* < 0.0001 from a Dunnett's post‐test examining the effect of parameters identified as significant in the two‐way ANOVA. (F, K) *p < 0.05, **** *p* < 0.0001 Dunnett's test post ANOVA. Data represented as mean ± SEM.

We randomized 6‐month‐old female and male 3xTg and non‐transgenic (NTg) control mice to one of four amino acid (AA)‐defined diets containing all 20 common AAs; the diet composition of the AA‐defined Control (Con) diet reflects that of a natural chow diet in which 21% of calories are derived from protein. The other three diets have a 67% reduction of isoleucine (IleR), leucine (LeuR), or valine (ValR); all diets were isocaloric with identical levels of fat, and the percentage of calories derived from AAs was kept constant by proportionally adjusting the amount of non‐essential AAs. These diets, which we have previously utilized [24], are detailed in Table S1. We tracked the mice longitudinally, tracking body weight monthly and assessing body composition at the start and end of the study, which spanned nine months. The experimental design is summarized in Figure 1A.

Both IleR‐fed and ValR‐fed female 3xTg and NTg mice maintained their body weight over the course of the study, while Control‐fed and LeuR‐fed females continued to gain weight (Figure 1B; Figure S1A). IleR‐ and ValR‐fed mice exhibited reduced fat mass and lean mass gain during the 9‐month study; by the end of the experiment, we observed an overall effect of diet on both lean and fat mass (Figure 1C,D; Figure S1B–D). At completion of the study, IleR‐ and ValR‐fed females had reduced adiposity compared to Control‐fed females in both genotypes (Figure 1E). These changes in body weight and body composition were not the result of reduced caloric intake; rather, as we have previously observed in other mouse strains, IleR‐fed females, irrespective of genotype, consumed more calories than Control‐fed females (Figure 1F; Figure S1E) [22]. We also observed increased food consumption in LeuR‐fed 3xTg mice, while ValR‐fed NTg females consumed less food.

The result of restricting the individual BCAAs on the weight and body composition of male 3xTg and NTg mice was similar, except that LeuR‐fed males had reduced accretion of fat mass and an overall reduction of adiposity (Figure 1G–J; Figure S1F–I). Surprisingly, and unlike in females, we did not observe a significant effect of any of the BCAAs on food consumption in either genotype, although food consumption was higher in IleR‐fed NTg mice (*p* = 0.0908) (Figure 1K; Figure S1J).

**FIGURE 2:**
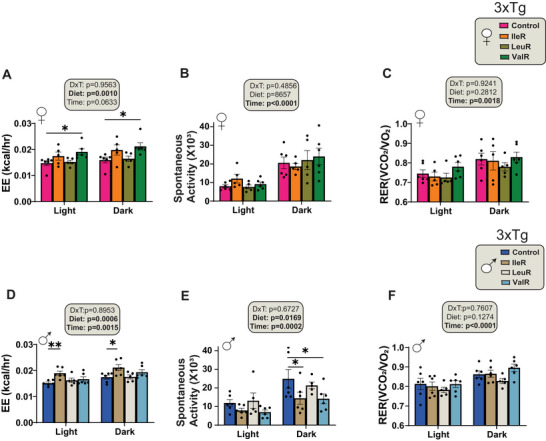
Distinct effects of individual BCAA restriction on energy balance in 3xTg mice. (A–F) Metabolic chambers were used to determine energy expenditure, spontaneous activity, and fuel source utilization over 24 h in female (A–C) and male (D–F) six‐month‐old 3xTg fed the indicated diets for 3 months. (A, D) Energy expenditure normalized to body weight in females (A) and males (D). (B, E) Spontaneous activity of females (B) and males (E). (C, F) Respiratory exchange ratio (RER) in females (C) and males (F). (A–C) *n *= 6 Con, *n *= 5 IleR, *n *= 5 LeuR and *n *= 6 ValR fed 3xTg biologically independent mice. (D–F) *n *= 6 Con, *n *= 6 IleR, *n *= 5 LeuR and *n *= 6 ValR fed 3xTg biologically independent mice. (A–F) Statistics for the overall effect of diet and time represent the *p*‐value from a two‐way ANOVA; *p < 0.05, **p < 0.01 from a Dunnett's post‐test examining the effect of parameters identified as significant in the two‐way ANOVA. Data represented as mean ± SEM.

Since IleR and ValR‐fed mice gained less weight than Control‐fed mice despite similar or increased food consumption, we examined the energy balance of all groups using metabolic chambers. Based on our previous findings, we expected that IleR and ValR‐fed mice would have increased energy expenditure [24]. Indeed, we found that a ValR diet significantly increased energy expenditure in 3xTg females, while IleR‐fed 3xTg female mice showed a non‐significant increase in energy expenditure (*p *= 0.1614, dark cycle) (Figure 2A). No differences in energy expenditure were observed with diet in NTg females (Figure S2A). In addition, no diet‐induced differences in activity level or the respiratory exchange ratio (RER), which is calculated using the ratio of O_2_ consumed and CO_2_ produced, were observed in either genotype of female mice (Figure 2B,C; Figure S2B,C).

In 3xTg males, food intake did not differ significantly across dietary groups, but IleR‐fed 3xTg male mice had significantly increased energy expenditure; there was also decreased activity in IleR‐fed and ValR‐fed males, without significant effects on RER (Figure 2D–F). Both IleR‐fed and ValR‐fed NTg males had increased energy expenditure, which was accompanied by higher activity levels in IleR‐fed NTg males only (Figure S2D,E). RER was not significantly different between groups (Figure S2F). Together, these findings suggest that BCAA restriction alters body composition primarily through changes in EE rather than food intake or substrate utilization, and that the magnitude of these metabolic shifts differs between 3xTg and NTg genotypes and by sex.

**FIGURE 3:**
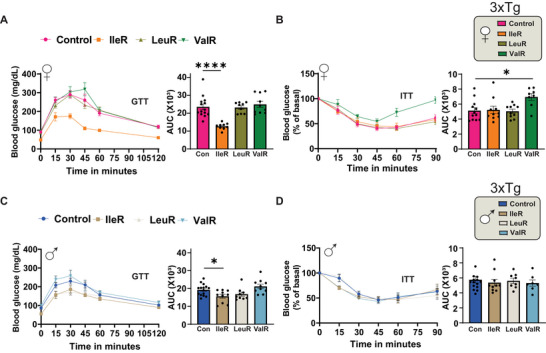
Isoleucine restriction improves glucose tolerance in 3xTg‐AD mice of both sexes. (A,B) Glucose (A) and insulin (B) tolerance tests were performed in female 3xTg mice fed the indicated diets for 3 months. (A) GTT: *n *= 14 Con, *n *= 9 IleR, *n *= 9 LeuR, and *n *= 10 ValR 3xTg biologically independent mice per group. (B) ITT: *n *= 12 Con, *n *= 9 IleR, *n *= 9LeuR and *n *= 9 ValR 3xTg biologically independent mice per group. (C,D) Glucose (C) and insulin (D) tolerance tests were performed in male 3xTg mice fed the indicated diets for 3 months. (A) GTT: *n *= 14 Con, *n *= 10 IleR, *n *= 9 LeuR, and *n *= 10 ValR fed 3xTg biologically independent mice (B) ITT: *n *= 14 Con, *n *= 10 IleR, *n *= 9 LeuR, and *n *= 8 ValR fed biologically independent mice. (A–D) *p < 0.05, ****p < 0.0001, Dunnett's test post ANOVA. Data represented as mean ± SEM. AUC, Area Under the Curve.

We previously showed that 3xTg mice have impaired glucose tolerance that is improved by PR in both sexes [15]. As such, we assessed the effect of restricting each BCAA on glycemic control by performing glucose (GTT) and insulin (ITT) tolerance tests at approximately 9 months of age, after the mice had been on their respective diets for about three months. In both female 3xTg and NTg mice, IleR feeding improved glucose tolerance (Figure 3A; Figure S3A); however, the ValR diet in 3xTg females and the IleR diet in NTg females impaired insulin sensitivity (Figure 3B; Figure S3B). In 3xTg and NTg males, a IleR diet also improved glucose tolerance, but there was no impact of diet on insulin sensitivity in 3xTg males; in NTg males, the IleR and ValR diets improved insulin sensitivity while a LeuR diet impaired insulin sensitivity (Figure 3C,D; Figure S3C,D).

### 2.2 Restriction of Specific BCAAs Improves Neuropathology in 3xTg Mice

We next assessed the effect of restriction of each BCAA on the progression of AD neuropathology by evaluating several pathological hallmarks of AD, including amyloid beta (Aβ) plaque deposition, phosphorylation of tau, and gliosis. Based on our previous characterization of this model [15], we examined AD neuropathology in 15‐month‐old female and male 3xTg mice, after 9 months of consuming either the Control or individual BCAA‐restricted diets.

**FIGURE 4:**
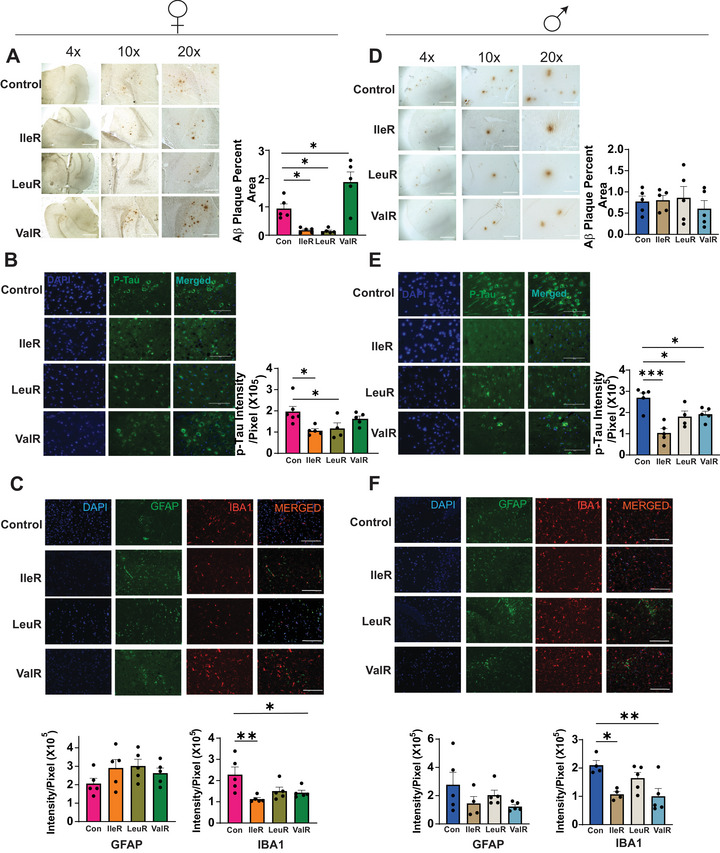
Restriction of isoleucine and leucine improves neuropathological outcomes in a sex dependent manner in 3xTg‐AD mice. (A–F) Analysis of AD neuropathology in female and male 3xTg mice fed the indicated diets from 6–15 months of age. (A, D) Representative plaque images of DAB staining with 6E10 antibody in the hippocampus of female (A) and male (D) 3xTg mice. 4x, 10x, 20x and 40x magnification shown; scale bar in the 4x image is 1000 µM, 10x image is 400µM, 20x is 200 µM and 40x is 100 µM. Quantification of plaque area in females shown under representative blots from hippocampus. (A, D) For hippocampus *n *= 4–5 3xTg biologically independent mice per group were used in both sexes. (B, E) Representative immunofluorescence images in the hippocampus of 3xTg females (B) and males (E) stained with p‐Tau Thr231 antibody (AT180), 40x magnification shown; scale bar 100 µM. Quantitative analysis of fluorescence intensity. *n *= 4–5 3xTg biologically independent mice per group. (C, F) Immunostaining and quantification of 5 µm paraffin‐embedded brain slices for astrocytes (GFAP) and microglia (Iba1) in female 3xTg mice (C) and male mice (F). 20x magnification shown; Scale bar is 200 µM. *n *= 4–5 biologically independent mice/group. (A–F) *p < 0.05, ** *p* < 0.01, ***p < 0.001, Dunnett's test post ANOVA. Data represented as mean ± SEM.

We observed substantial Aβ plaque accumulation in the hippocampus of Control‐fed 3xTg females; IleR‐fed and LeuR‐fed mice had substantially reduced plaque deposition, while ValR‐fed mice had double the number of plaques as Control‐fed females (Figure 4A). Fluorescent immunostaining and quantitative analysis of hippocampal phosphorylated tau (p‐Tau Thr231) revealed that IleR and LeuR‐fed females had significantly reduced Tau phosphorylation (Figure 4B). At the level of the whole brain, we observed a significant reduction in p‐Tau Thr231 only in ValR‐fed females (Figure S4A). Finally, neuroinflammation is a key pathological feature of AD, and we assessed activation of astrocytes and microglia. We conducted immunostaining of brain sections with anti‐glial fibrillary acidic protein (GFAP), an astrocyte marker, and with anti‐ionized calcium binding adaptor molecule 1 (IBA‐1), a microglia marker. While there was no effect of diet on astrocytic activation, restriction of any BCAA reduced microglial activation, reaching significance in the case of IleR‐ and ValR‐fed females (Figure 4C).

In 3xTg males, Aβ plaque deposition was less prominent than in females, and there was no effect of restricting any of the BCAAs (Figure 4D). However, restriction of any BCAA in males significantly reduced hippocampal p‐Tau Thr231 as shown by fluorescent immunostaining (Figure 4E). At the whole brain level, there was no effect of IleR or LeuR diets on p‐Tau, but ValR‐fed males had a significant increase in p‐Tau (Figure S4B). While there was no effect of diet on astrocytic activation, restriction of either Ile or Val, but not Leu, significantly reduced microglial activation in the hippocampus, as shown by reduced IBA‐1 expression (Figure 4F).

### 2.3 Transcriptomic Profiling of the Brain Reveals Sex‐Specific and Overlapping Molecular Responses to Individual BCAA Restriction in 3xTg Mice

**FIGURE 5:**
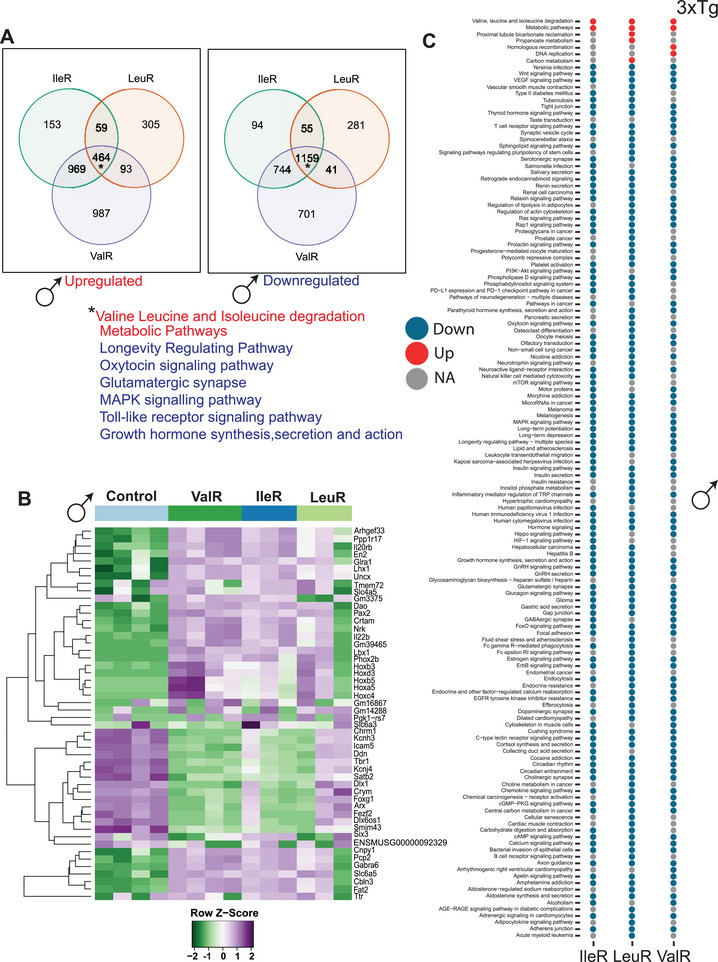
Transcriptional profiling of the brain identifies overlapping molecular responses to individual BCAA restriction in male 3xTg mice. (A) Venn diagram of the upregulated and downregulated differentially expressed genes in the three BCAA‐restricted groups. * The pathways listed below the Venn diagram represent a subset of significantly enriched KEGG pathways (*p* < 0.05) from differentially expressed genes altered by all three BCAA‐restricted diets. (B) Heatmap of the top 50 differentially expressed (DEG) genes (C) Enriched transcriptomic pathways across all male groups (red = upregulated, blue = downregulated, grey = not significant). *n *= 4–5 mice/group.

To obtain insight into the molecular pathways altered by individual BCAA restriction, we next performed transcriptomic analysis of the whole brain of both male and female 3xTg mice fed the Control, IleR, LeuR, or ValR diets. We identified the differentially expressed genes (DEGs) expressed as a result of restricting each of the BCAAs. Interestingly, we identified a shared set of 454 upregulated and 1159 downregulated DEGs that were differentially expressed across all three BCAA‐restricted groups in 3xTg males (Figure 5A; Table S2). Restriction of each individual BCAA also resulted in DEGs that were unique to that group, with ValR‐fed males showing the largest number (1,688) of DEGs vs. Control‐fed males, which were not shared with either LeuR or IleR‐fed males. We failed to observe any DEGs shared across the three restricted diets in female 3xTg mice (Figure S5 and Table S3).

Analyzing the expression of the 50 most variable genes revealed shared and distinct gene expression patterns across diet groups. Among the differentially expressed genes, several Hox homeobox genes (*Hoxb3, Hoxa5, Hoxc4, Lhx1*), which have been shown to play critical roles in neurodevelopment and synaptic plasticity and have been implicated in AD pathology [31, 32], showed altered expression across all the restricted diets in male 3xTg mice (Figure 5B). Consistent with the distinct gene expression patterns observed across all the individual BCAA diet groups, we also found that *Foxg1* (Forkhead box G1), which has been implicated in neuronal development [33], was downregulated in ValR and IleR‐fed males; however, expression in LeuR‐fed males was similar to that of Control‐fed males. Furthermore, we found that *Satb2*, a gene known for its role in neurodevelopment and cognition, was downregulated in both ValR and IleR‐fed males [34]. We also found that *Crym*, which encodes the neuroprotective protein µ‐crystallin, was downregulated in ValR and IleR‐fed males [35, 36].

We next performed pathway enrichment analysis to identify biological pathways altered in response to BCAA restriction. In male 3xTg mice, we observed substantial overlap in the pathways across all three restricted BCAA groups, with most pathways downregulated in two or more groups (Figure 5C). Although most of the pathways affected by BCAA restriction were downregulated, a few were upregulated, with “Valine, Leucine, and Isoleucine Degradation” and “Metabolic Pathways” consistently upregulated in all the restricted groups (Figure 5C; Table S4). These upregulated pathways could reflect the compensatory metabolic adaptation in the brain occurring due to the limited intake of BCAAs. “Carbon Metabolism & Propanoate Metabolism” was upregulated exclusively in LeuR mice, perhaps suggesting the increased need for alternate carbon sources to meet the metabolic demands caused by leucine restriction. “DNA Replication” was selectively upregulated in ValR‐fed mice, which could suggest increased cellular activity or stress response, possibly reflecting reactive glial cell activation or DNA repair mechanisms [37]. As ValR males also exhibited worsened AD pathology, specifically increased p‐tau in the whole brain, this change might represent a pathological cellular response.

Most overlapping pathways were downregulated. Neuroinflammatory pathways were downregulated across all three BCAA‐restricted groups, including “MAPK signaling pathway” and “Toll‐like receptor signaling pathway,” potentially contributing to the observed cognitive improvements and reduced microglial activation. In addition, the “Longevity‐regulating pathway” and “Growth hormone synthesis and secretion” pathways were downregulated in all three restricted groups, suggesting a potential reduction in growth and aging‐related signaling. It was particularly interesting to see the downregulation of “mTOR signaling pathway” only in the IleR group, as we had expected to see this in the LeuR group, as leucine, but not isoleucine, is a very potent mTOR agonist. We also observed LeuR‐specific downregulation of pathways, including “pathways in neurodegeneration” and “cellular senescence pathways”. In contrast, “Pathways in cancer” was downregulated only in IleR and ValR mice.

Together, these findings indicate that while individual BCAA restriction induces distinct molecular effects, they also engage overlapping molecular mechanisms in males. These shared molecular mechanisms regulate pathways involved in neuroinflammation as well as metabolism and likely contribute to the ability of individual BCAA restriction to delay the progression of AD pathology in males.

### 2.4 Circulating and Brain BCAA Levels are Maintained in 3xTg Mice Following Long Term Restriction of Individual BCAAs

To determine whether dietary restriction of each individual BCAA altered systemic or brain amino acid availability, we quantified serum and brain BCAA abundance using LC/MS after 9 months of dietary intervention in the 3xTg mice. Despite a 67% reduction in dietary isoleucine, leucine, or valine, we found no significant alterations in the plasma or tissue levels of any of these BCAAs in 3xTg males or females (Figure S6A–D). While these findings may appear surprising, they are consistent with our previous observations that long‐term restriction of BCAAs does not lead to reduced plasma levels of BCAAs [22, 24, 38], and are consistent with the ability of mice to buffer blood levels of amino acids against feeding [39]; and the plasma levels of BCAAs and other amino acids is the major determinant of BCAA transport across the blood brain barrier [40].

### 2.5 Isoleucine Restriction Downregulates Autophagy and Reduces mTORC1 Activity in 3xTg Mice

We were surprised that autophagy was not implicated as an altered pathway in our transcriptional analysis, and that mTOR signaling was identified as altered only in IleR‐fed mice. Both autophagy and mTOR are heavily implicated in the pathogenesis of AD; impaired autophagy machinery leads to ineffective clearance of plaques and tangles, contributing to the progression of AD symptoms in both patients and animal models [41, 42, 43, 44, 45], while hyperactivation of mTOR in AD disrupts the proteostasis network and acts to inhibit autophagy, exacerbating the accumulation of plaques and tangles [46]. Inhibiting mTOR, either pharmacologically with the drug rapamycin or via feeding of a PR diet, promotes autophagy and reduces AD pathology [15, 47, 48, 49, 50]. We therefore further investigated the effect of individual BCAA restriction on autophagy and mTOR signaling at the protein level.

**FIGURE 6:**
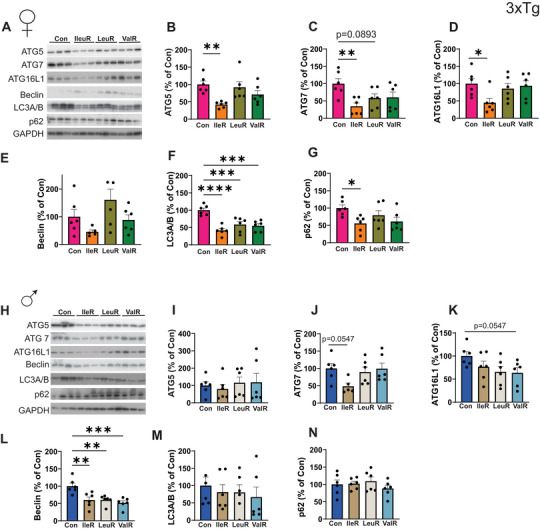
Distinct effects of individual BCAAs on autophagy in 3xTg‐AD mice. (A–N) Immunoblotting on brain lysates of 15‐month‐old 3xTg mice fed the indicated diets to assess several autophagy markers, including autophagy proteins ATG5, ATG7, and ATG16L1, as well as autophagosome formation proteins Beclin and light chain 3A/B (LC3A/B), and the autophagy receptor p62 (sequestosome 1, SQSTM1). (A) Representative immunoblot of all the autophagy‐related proteins in females. (B–G) Quantification of ATG5 expression (B), ATG7 (C), ATG16L1 (D), Beclin (E) LC3A/B (F), and p62 (G) relative to expression of GAPDH in females (B–G) *n *= 6 3xTg biologically independent mice per group. (H) Representative immunoblot of all the autophagy related protein in males. (I–N) Quantification of ATG5 expression (I), ATG7 (J), ATG16L1 (K), Beclin (L) LC3A/B (M) and p62 (N) relative to expression of GAPDH in males (I–N) *n* = 6 3xTg biologically independent mice per group. (B–G, I–N) *p < 0.05, **p < 0.01, ***p < 0.001, ****p < 0.0001, Dunnett's test post ANOVA. Data represented as mean ± SEM.

We performed immunoblotting on brain lysates to assess several autophagy markers, including autophagy proteins ATG5, ATG7, and ATG16L1, as well as autophagosome formation proteins Beclin and light chain 3A/B (LC3A/B), and the autophagy receptor p62 (sequestosome 1, SQSTM1). In female 3xTg mice, IleR‐fed mice exhibited significantly reduced expression of ATG5, ATG7, LC3A/B, and p62 compared to Control‐fed mice. LeuR and ValR‐fed females showed a significant decrease in LC3A/B expression, a key marker of autophagosome formation, but no significant changes in any other markers (Figure 6A–G). In 3xTg males, we observed a significant downregulation of Beclin in all three BCAA‐restricted groups compared to control‐fed mice. Although certain other autophagy markers exhibited a decreasing trend in IleR and ValR‐fed mice, none of these changes reached statistical significance (Figure 6H–N).

We next evaluated mTORC1 activity by performing immunoblotting for the phosphorylation of its substrates, p‐S240/S244 S6 and T37/S46 4E‐BP1, in whole brain lysates of both female and male mice (Figure S7A–F). In female 3xTg mice, none of the individual BCAA restricted diets led to significant changes in phosphorylation of S240/S244 S6 compared to the Control‐fed mice. However, phosphorylation of T37/S46 4E‐BP1 was significantly reduced in IleR‐fed and LeuR‐fed 3xTg females compared to Control‐fed mice (Figure S7A–C). In 3xTg males, restriction of any of the three BCAAs resulted in reduced phosphorylation of p‐S240/S244 S6 as compared to Control‐fed mice (Figure S7D,E). However, while ValR‐fed 3xTg males had reduced phosphorylation of T37/S46 4E‐BP1 relative to Control‐fed mice, IleR‐fed 3xTg males unexpectedly had increased T37/S46 4E‐BP1 phosphorylation compared to Control‐fed mice (Figure S7F).

### 2.6 Sex‐Specific Benefits of Individual BCAA Restriction on Hippocampal‐Dependent Spatial Learning‐Associated Memory Deficits

To evaluate the effects of individual BCAA restriction on cognition, we conducted behavioral assays on 12‐month‐old 3xTg and NTg mice fed either a Control, IleR, LeuR, or ValR diet. We examined the performance of the animals in a Barnes Maze and tested Novel Object Recognition (NOR).

**FIGURE 7:**
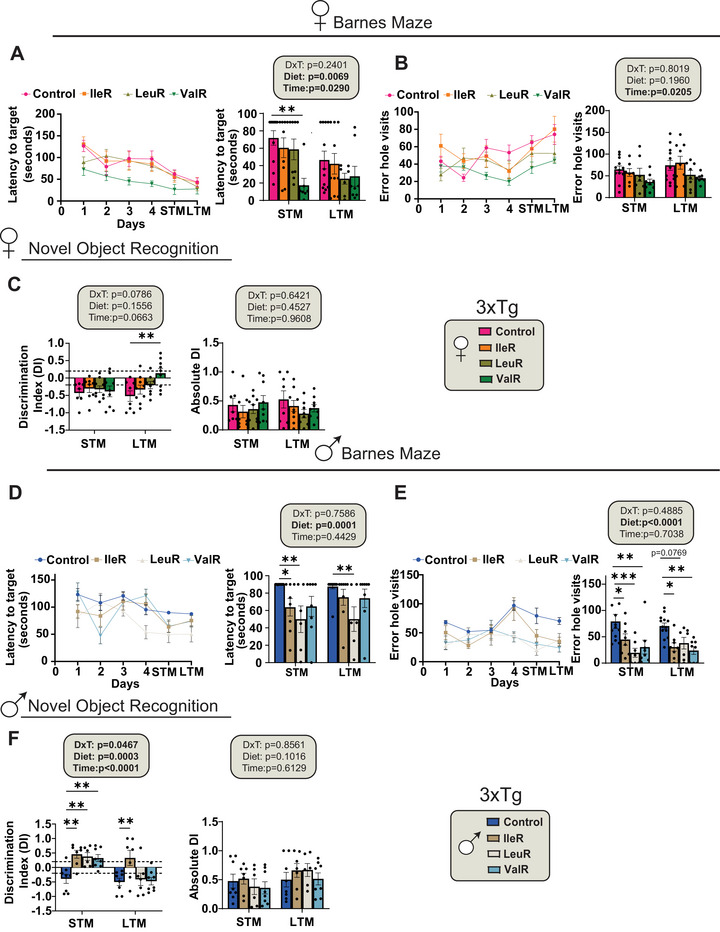
Valine restriction improves cognition in females, while Isoleucine and Leucine restriction improve memory in males. (A–F) The behavior of female and male 3xTg‐AD mice was examined at 12 months of age after mice were fed the indicated diets for 6 months. (A, D) Latency of target in Barnes Maze acquisition period over the five days of training and in short‐term memory (STM) and long‐term memory (LTM) tests in female (A) and male mice (D). (B, E) The number of error hole visits during the Barnes maze training phase in STM and LTM tests by female (B) and male (E) mice. (C, F) The preference for a novel object over a familiar object was assayed in female (C) and male (F) mice via STM and LTM tests. The dashed lines at +0.2 and −0.2 indicate the threshold for discrimination index (DI) values showing the preference for novel or familiar objects. Absolute DI was plotted to show the magnitude of discrimination regardless of the direction of preference. (A,B) *n *= 12 Con, *n *= 10 IleR, *n *= 8 LeuR, and *n *= 8 ValR fed biologically independent 3xTg female mice. (C) *n *= 8–9 3xTg biologically independent 3xTg females per group. (D,E) *n *= 15 Con, *n *= 8 IleR, *n *= 7 LeuR, and *n *= 8 ValR fed biologically independent 3xTg male mice. (C) *n *= 8 3xTg biologically independent 3xTg males per group. (A–F) Statistics for the overall effects of diet, time, and the interaction represent the *p*‐value from a two‐way ANOVA. *p < 0.05, **p < 0.01, ***p < 0.001, ***p < 0.0001, Dunnett's post‐test examining the effect of parameters identified as significant in the two‐way ANOVA. Data represented as mean ± SEM.

In the Barnes maze, mice were required to locate an escape box placed at the target hole using spatial cues learned during a four‐day acquisition phase. Short‐term memory (STM) and long‐term memory (LTM) were tested on days 5 and 12, respectively. Among the female 3xTg mice, ValR‐fed females located the escape box the fastest during the training phase and showed the best performance during both the STM and LTM tests, taking significantly less time to identify the escape hole during the STM test (Figure 7A). No significant differences were observed in error hole visits across all groups during training sessions or the STM and LTM tests, but consistent with their improved latency, ValR‐fed females tended to make fewer errors than mice fed the Con diet (Figure 7B). As expected, we did not observe any significant cognitive deficits in age‐matched NTg female mice following STM and LTM in the BM test (Figure S8A,B).

NOR tests the preference for investigating a familiar object vs. a new object and is quantified based on a discrimination index (DI) following a STM and LTM test. The DI is calculated as the difference in time spent exploring the novel versus the familiar object, divided by the total exploration time of both objects. A positive DI implies a preference for exploring novelty, indicating that the familiar object's memory persists and the mice favor exploring the new object. Conversely, a negative DI shows that the mice spent more time exploring the familiar object. In both the STM and LTM tests, the 3xTg females on IleR and LeuR diets consistently had negative DI; thus, these mice failed to explore novel objects and spent more time with the familiar object; ValR‐fed females similarly had a negative DI on the STM test (Figure 7C). On the LTM test, ValR‐fed females showed a neutral DI, indicating they were unable to discriminate between objects; however, these animals still had significantly greater NOR than Control‐fed females (Figure 7C). We also plotted absolute DI to see the overall strength of discrimination irrespective of whether it was novel or familiar, and we did not see any significant changes between any groups in both STM and LTM (Figure 7C). NTg female mice fed the LeuR diet spent less time exploring the novel object and hence had a significantly negative DI as compared to Control‐fed NTg females in both the STM and LTM tests (Figure S8C).

In contrast to females, LeuR‐fed male 3xTg mice showed significant improvements in Barnes maze latency to the target during both the STM and LTM tests, and IleR‐fed male 3xTg mice showed significantly improved latency to the goal during the STM test only (Figure 7D). This was associated with a decrease in errors, with IleR, LeuR, and ValR‐fed 3xTg males having fewer errors during both the STM and LTM tests compared to Control‐fed mice (Figure 7E). In the NOR test, all three BCAA‐restricted groups of 3xTg males had a positive DI that was significantly greater than the negative DI of the Control‐fed mice during the STM trial, demonstrating improved recognition memory (Figure 7F, left). In the LTM trial, only IleR‐fed males showed a positive DI, which was significantly greater than that of the Control‐fed males, again demonstrating improved memory. When we assessed the magnitude of discrimination by plotting absolute DI, all groups were statistically equivalent (Figure 7F, right). We did not observe any cognitive deficits in age‐matched NTg males, and thus it was not surprising that we did not observe that any of the restricted diets improved NOR or Barnes maze performance (Figure S8D–F).

Finally, after completing the metabolic, behavioral, and molecular phenotyping, we visualized the global effects of individual BCAA restriction on overall phenotypic traits. We performed Principal Component Analysis (PCA) using a combined phenotypic data set including 26 traits, including metabolic, behavioral, and pathological data. In 3xTg males, PCA showed distinct clustering between the Control, IleR, LeuR, and ValR groups, with the greatest separation shown between Control‐fed and ValR‐fed groups (Figure S9A,B). Looking at the key variables, we found hippocampal plaque load, energy expenditure (light and dark), and RER (dark) as some of the strongest contributors to the second principal component (Component 2) driving the separation. In female 3xTg mice, PCA also showed clear separation between the diet groups, particularly between Control‐fed mice and mice fed either the ValR or IleR diets (Figure S9C). The strongest contributors to the first principal component (Component 1) driving this separation in females were end body weight, lean, and fat mass (Figure S9C,D).

### 2.7 IleR Improves the Survival of Male 3xTg Mice

**FIGURE 8:**
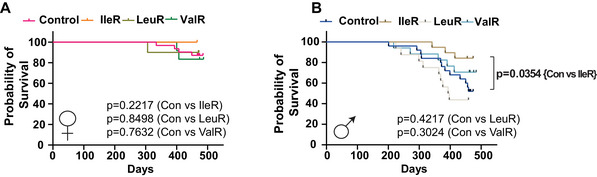
Isoleucine restriction improves survival in male 3xTg‐AD mice. (A,B) Kaplan–Meier plots of the survival of female (A) and male (B) 3xTg female mice fed the indicated diets starting at 6 months of age. (A) For females: *n *= 30 Con, *n *= 10 IleR, *n *= 10 LeuR, and *n *= 12 ValR fed 3xTg biologically independent mice. (B) For males: *n *= 21 Con, *n *= 20 IleR, *n *= 15 LeuR, and *n *= 15 ValR fed 3xTg biologically independent mice. *p *= 0.0345, log‐rank test Con vs. IleR mice.

Previous studies, including our own, have reported high mortality rates in male 3xTg‐AD mice, and we have shown that PR promotes survival of these animals [15, 51, 52]. Although female 3xTg mice had low mortality and no differences were observed between diet groups, Control‐fed 3xTg males had high mortality, which was significantly reduced by IleR (log‐rank test, Control vs. IleR, *p* = 0.0354) (Figure 8A,B). ValR‐fed mice also trended toward increased survival, while, surprisingly, LeuR‐fed mice had the worst survival outcomes.

### 2.8 Gene Co‐Expression Network Analysis Identifies Modules Associated With Metabolic and Cognitive Traits in Males

**FIGURE 9:**
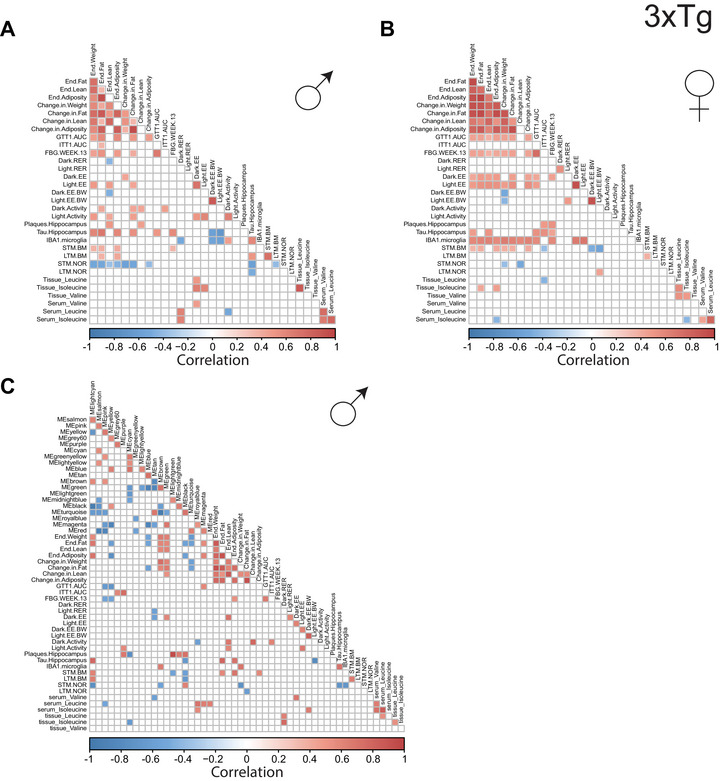
Correlation analysis identifies sex‐specific association of AD pathology, cognition, and gene expression. (A,B) Heat maps showing the Pearson correlation of the rankZ‐transformed traits of male (A) and female (B) 3xTg mice. (C) Pearson correlation of rankZ‐transformed traits and WGCNA module eigengenes (ME) in 3xTg male mice. (A–C) Color indicates the strength and directionality of the correlation, with red indicating a positive correlation and blue a negative correlation. Only statistically significant correlations (*p* < 0.05) are shown.

We performed correlation analysis of traits separately for each sex of 3xTg mice, calculating Pearson's correlation coefficients for rank‐ordered traits, and generating a heatmap to illustrate trait‐trait relationships (Figure 9A,B). In 3xTg males, we observed a significant negative correlation between hippocampal levels of Tau and both STM and LTM during NOR testing; there was also a positive association with latency to target during LTM Barnes maze testing (Figure 9A). Tau levels were also associated with microglial activation in males. Thus, we performed Weighted Gene Co‐Expression Network Analysis (WGCNA) to generate gene co‐expression networks to identify gene modules associated with metabolic, cognitive, and AD‐related traits in the brains of male 3xTg mice. These modules were assigned different colors for classification. We then correlated the module eigengene (ME) for each distinct gene module with other module MEs and rank‐ordered traits as illustrated in Figure 9C. We identified two gene modules, Lightcyan and Black, which were correlated with both hippocampal Tau and cognitive performance; we also identified the Lightgreen and Black gene modules as they correlated with hippocampal plaques and cognitive performance (Figure 9C and Table S5).

**FIGURE 10:**
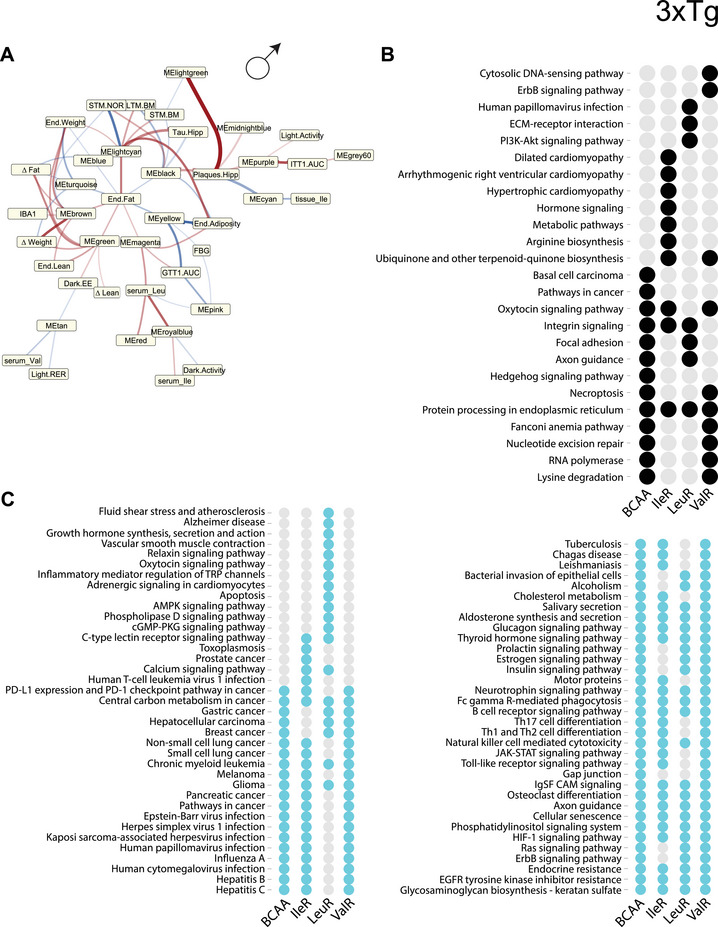
Gene modules enriched for metabolic and signaling pathways are strongly connected to AD pathology and cognitive function in 3xTg males. (A) ME‐trait correlations were used to construct a correlation network in Cytoscape. Color indicates direction of correlation (red = positive, blue = negative), thickness is controlled by |correlation| (range, 0.5 to 1.0), and transparency is set by the −log10 P‐value (range, 1 to 5). (B) Dot plot summarizing KEGG pathway enrichment analysis of genes within the Black module. (C) Dot plot summarizing KEGG pathway enrichment analysis of genes within the Lightcyan module. (B,C) Colored dots indicate that the pathway is significantly enriched (*p* < 0.05), genes were included if they were present in the module (Black or Light cyan) and were significantly differentially expressed (BH adjusted *p *< 0.05) when fed the indicated diet (IleR, LeuR, ValR) or genes differentially expressed in mice fed any of the diets (BCAA) as compared to Control‐fed mice.

We used ME‐trait correlations to construct a network in Cytoscape (Figure 10A; Table S6) where the nodes are MEs or physiological traits and edges represent the correlation between nodes. We found that the Lightcyan and Black modules formed hub nodes that were highly interconnected with cognitive performance, AD pathology, and aspects of metabolism, most notably weight, fat mass, and adiposity (Figure 10A). KEGG pathway analysis of the Black module (Figure 10B; Table S7) identified a number of pathways altered in all of the BCAA restricted diets, including “Hedgehog signaling pathway,” “Lysine degradation,” and “Necroptosis,” that have been implicated in neurodegeneration and cell death, in response to both Aβ and Tau pathology [53, 54, 55, 56]. Interestingly, we also identified BCAA‐specific alterations in signaling pathways (Figure 10B), with leucine restriction associated with changes in “PI3K‐Akt signaling pathway,” which is upstream of mTOR signaling and a key regulator of metabolism and aging; isoleucine and valine restriction were associated with altered “Oxytocin signaling”, which has been linked to AD [57]; and isoleucine restriction was associated with “Integrin signaling” [58]. Valine restriction was associated with “ErbB signaling pathway” and “Cytosolic DNA‐sensing pathway,” which have also been associated with AD [59, 60].

Similarly, both BCAA‐shared and BCAA‐specific KEGG pathways were identified in the Lightcyan module (Figure 10C and Table S8), including pathways associated with cancer, infection, and the immune system. There were also multiple hormonal signaling pathways and metabolic pathways implicated, including “Estrogen signaling pathway,” “Insulin signaling pathway,” “Glucagon signaling pathway,” and HIF‐1, Ras, and ErbB signaling. Cellular senescence, which we have recently shown is regulated by dietary BCAAs [61] and has been suggested as a driver of AD, was also altered in this module [62]. Leucine restriction induced alterations in the KEGG pathway “Alzheimer disease” as well as multiple other pathways, including the “AMPK signaling pathway” which is associated with AD in mice and humans [63, 64]. A few pathways in this module were specifically altered by IleR alone, while no pathways in this module were ValR‐specific.

In conclusion, we have identified specific gene modules associated with AD pathology and cognition that are enriched in genes involved in multiple AD‐related pathways. While there were many similarities in the pathways induced by restriction of any of the three BCAAs, restriction of each BCAA also had distinct transcriptional activity patterns that could mediate many of the BCAA‐specific effects we observed on metabolism, AD pathology, and cognition in males.

## 3 Discussion

Dietary protein is a key regulator of metabolic health in rodents and humans. A major contributor to the benefits of a low‐protein diet is the restriction of branched‐chain amino acids (BCAAs), particularly isoleucine, which has been shown to recapitulate many of the metabolic advantages of PR [24]. Restriction of protein, BCAAs, or isoleucine not only extends lifespan but promotes favorable molecular changes, rejuvenating the liver transcriptome, improving hepatic aging rate indicators, and reducing hepatic cellular senescence [13, 22, 23, 61]. Interventions that slow aging should be effective in the prevention or treatment of age‐related disease. In support of this hypothesis, we recently demonstrated that PR enhances cognitive function and delays the progression of AD pathology in 3xTg mice, and others have identified benefits of BCAA restriction for AD in this same mouse model [15, 21].

However, a critical question remained unanswered – do all BCAAs contribute equally to these benefits, or do they have distinct roles in mitigating AD progression? Here, we examined the effect of individually restricting leucine, isoleucine, or valine on AD progression using the 3xTg mouse model of this disease. We initiated the study at 6 months of age, an age at which 3xTg mice show cognitive deficits as well as aspects of AD pathology. We discovered that each of the BCAAs has a unique and sex‐specific effect on metabolic health, AD neuropathology, and cognitive performance, as summarized in Table S10. Strikingly, while ValR significantly improved cognitive function in females, it did not in males; conversely, IleR and LeuR enhanced cognition in males but not in females.

Interestingly, these cognitive improvements occurred independently of reductions in Aβ plaque burden in either of the sexes. Indeed, restricting valine increased the plaque deposition in the hippocampus of female mice, yet a ValR diet was still able to improve memory in female mice relative to females on a Control‐fed diet. In fact, ValR‐fed mice had the most pronounced memory improvements in females, further demonstrating that pathological burden may not always align with cognitive functioning. This observation is consistent with findings from clinical trials, where drug candidates targeting amyloid pathways have yielded more modest cognitive improvements than initially hoped, despite reducing amyloid burden [3, 65, 66, 67, 68, 69].

Given the limited correlation between plaque burden and cognitive outcomes, we considered other pathological features that could better explain the cognitive benefits of BCAA restriction. Indeed, we observed that improved cognitive function tended to correlate with reductions in tau pathology. Cognitive improvements following IleR‐ or LeuR‐feeding occurred in parallel with reduced hippocampal tau phosphorylation, indicating a close alignment with tau pathology and behavioral outcomes in males. While this was not observed in ValR‐fed females, which also showed improved cognitive function, these mice did show a reduction in whole‐brain tau phosphorylation. In our WGCNA analysis of 3xTg males, we found that tau phosphorylation was associated with both worse metabolic outcomes – higher weight and adiposity, worse glycemic control – as well as decreased cognitive function. These findings suggest that mechanisms beyond classical amyloid accumulation that are linked with tau pathology, as well as organismal metabolism, may play a key role in mediating the cognitive benefits of BCAA restriction.

Beyond neuropathological changes, BCAA restriction also affected systemic metabolism. Metabolic dysfunction is a hallmark of AD, with insulin resistance and impaired glucose metabolism contributing to neuronal energy deficits [70, 71]. Recent studies have identified elevated plasma BCAA levels, particularly isoleucine, in AD patients, suggesting a possible link between BCAA dysregulation and disease progression [18, 19, 72]. Consistent with these findings, our study demonstrates that restricting isoleucine and valine had the most beneficial effects on body weight and adiposity in females and males, whereas leucine restriction had a detrimental impact, leading to increased adiposity in both sexes.

Importantly, our findings offer new mechanistic insights into how BCAA restriction influences AD progression. Despite worsening insulin sensitivity and increased plaque formation, valine restriction emerged as one of the most significant BCAAs in improving cognition in females. Moreover, this improvement in cognition occurred independently of changes in insulin sensitivity, neuroinflammation, mTORC1 signaling, or autophagy activation in the brain, aligning well with the Cognitive Reserve hypothesis [73, 74], which suggests that there can be a mismatch between pathological burden and cognitive performance. Furthermore, while mTORC1 inhibition and autophagy activation are traditionally linked to neuroprotection, our findings suggest that cognitive improvements can arise through mechanisms distinct from these pathways, such as enhanced synaptic plasticity, altered neuroimmune signaling, or improved neuronal connectivity.

Previous studies show that cognitive performance in AD models correlates more strongly with synaptic plasticity than amyloid burden [75, 76, 77, 78], and long‐term potentiation (LTP) deficits can be modified independently of plaque/tangle burden by dietary interventions [79, 80, 81, 82]. Neuroinflammatory signaling also significantly influences cognitive outcomes independent of plaques [83, 84, 85]. These findings are consistent with our recent CR study showing cognitive benefits dissociated from Aβ pathology [4].

In contrast, IleR‐ and LeuR‐fed males showed both cognitive improvements and reduced hippocampal tau, suggesting tau reduction partly mediates their cognitive benefits and that tau may have a stronger impact on cognitive decline than amyloid burden in males. Importantly, tau changes were region‐specific. Hippocampal phospho‐tau (Thr231) was significantly reduced across all BCAA‐restricted groups, while whole brain p‐tau remained largely unchanged in IleR and LeuR‐fed mice and elevated in ValR‐fed males, consistent with the 3xTg model's pronounced hippocampal tau accumulation [29, 86, 87, 88]. Whole‐brain homogenates likely dilute these localized effects. Future studies using electrophysiological recordings, synaptic protein analysis, and neuroinflammation profiling will help us to determine the mechanisms underlying these sex and diet‐specific cognitive effects.

Impaired autophagy is a well‐recognized contributor to the dysregulation of Aβ metabolism in AD (Nilsson and Saido 2014, Uddin et al. 2018). In our study, IleR led to a reduction in plaque deposition in both males and females, accompanied by decreased microglial activation as shown by reduced IBA1 expression. However, quite surprisingly, most autophagy‐related proteins and autophagosome formation markers were downregulated in females from the IleR group, with little to no change in males as well. This may suggest that an alternate mechanism, potentially involving the ubiquitin–proteasome system (UPS) or enhanced phagocytic clearance, may have played a significant role under BCAA restriction [89]. However, this remains a hypothesis, and additional studies will be required to determine whether UPS activation or another mechanism mediates these effects. Another possibility is that autophagy was less activated due to a reduced burden of misfolded or aggregated proteins, leading to lower demand for degradation pathways.

In line with these findings, transcriptomic analysis of the brain across all three BCAA‐restricted groups identified several distinct and overlapping molecular pathways that correlated with metabolic, behavioral, and neuropathological outcomes. These pathways were mostly observed in males rather than females. Interestingly, we observed many downregulated pathways with very few pathways being upregulated across all groups in 3xTg males. The “Metabolic Pathways” and “Valine, leucine, and isoleucine degradation pathways” were upregulated in all three individual BCAA‐restricted groups. The upregulation of these pathways might suggest a compensatory metabolic adaptation to reduced BCAA intake for maintaining energy demands. Our WGCNA analysis identified gene modules associated with cognition and AD pathology that highlight known AD‐related pathways, such as the insulin/PI3K/AKT/mTOR/AMPK pathway, as well as a number of metabolic and hormonal processes that are less appreciated for their involvement in AD. Further investigation of these pathways and processes for their involvement in AD, as well as the response of AD mice to dietary reduction in BCAAs, is likely warranted.

Despite reducing dietary intake of each individual BCAA by 67%, we did not observe any significant reductions in circulating or brain BCAA levels in either sex of 3xTg mice. This is consistent with our previous studies, in which we have not observed decreases in plasma BCAAs following this degree of restriction [22, 24, 38], as well as similar findings in protein‐restricted humans [10]. Notably, this occurs despite protein restriction substantially lowering portal vein levels of amino acids [39] and suggests that plasma levels of BCAAs, as well as other amino acids, are tightly regulated, analogous to the regulation of blood sugar. Brain levels of amino acids transported by the Large Amino Acid Transporter (LAT1) are primarily mediated by the ratio of these amino acids in plasma. The exact mechanism by which the brain senses a decrease in dietary BCAAs is unknown but could involve direct sensing of BCAA levels in the gut or portal vein, or indirectly via changes in circulating metabolites; this latter possibility would suggest that the beneficial effects of BCAA restriction may be mediated through changes in hormones or other metabolites rather than through alterations in tissue BCAA levels.

Limitations of the present study include the exclusive use of the 3xTg‐AD mouse model; the use of other AD mouse models could give improved insight, particularly into understanding if the effects of individual BCAAs are mediated by their effects on Aβ, tau, or both. Further, as different strains of mice have different metabolic responses to BCAAs, the effects of BCAA on AD development and progression may vary because of genetic background. While we have summarized genotype‐dependent and independent effects on various phenotypes in Table S11, our recent work demonstrates that isoleucine restriction produces robust metabolic benefits across different sexes and strains (C57BL/6J and DBA/2J); however, molecular responses reveal many sex and strain‐dependent pathway changes, suggesting that findings may not be universally conserved across genetic backgrounds [38].

Due to the age‐associated mortality and the multiple groups of mice studied, cohort sizes, particularly for analyses of pathology, were modest, although generally aligned with those used in other long‐term studies of 3xTg mice by our group and others [4, 15, 90]. However, the consistency of our findings across metabolic, behavioral, and molecular markers all support our conclusions despite the limited cohort size.

Our molecular analyses were fairly limited. Our study focused on a single phosphorylation site of tau, Thr231, previously implicated in AD pathology [91, 92]. However, multiple tau phosphorylation sites contribute to AD pathology, and the effect of BCAA on these sites could be examined in future studies. Our molecular analyses primarily concentrate on changes in the whole brain; a more extensive and comprehensive analysis will be necessary to elucidate the intricate molecular mechanisms engaged by BCAAs within the different regions of the brain. Additionally, even though we examined autophagy‐related proteins, we did not directly assess autophagic flux, which will be beneficial for distinguishing between autophagy initiation and impaired degradation.

Finally, our study was not designed to differentiate acute versus chronic effects of BCAA restriction. Prior work from our lab in multiple strains of mice has shown that short‐term restriction of BCAAs or isoleucine or valine alone can induce FGF21, increase energy expenditure and alter fuel utilization, and remodel the hepatic transcriptome. Whether the sex‐specific benefits to memory and AD pathology observed here can be induced acutely in late life in animals with developing AD pathology or are only observed when mice are placed on these diets chronically prior to the onset of AD will provide critical insights into the mechanism and impact the feasibility of translating these types of dietary interventions to humans.

In summary, we have shown that restricting each of the BCAAs has both unique and distinct effects on AD progression in 3xTg mice. Restricting dietary isoleucine and valine broadly improves metabolic health in both sexes, while restricting leucine has negative impacts on overall metabolic health. While restricting isoleucine and leucine were both beneficial in suppressing AD pathology, valine restriction worsened the pathological outcomes, primarily in females. Despite this, ValR females exhibited significant cognitive improvements, suggesting that cognitive benefits may occur independently of AD pathology. In contrast, IleR and LeuR had a more pronounced effect on memory in males. On a molecular level, we observed sex and diet‐specific effects of individual BCAA restriction on mTORC1 signaling, autophagy, and gene expression. Finally, restricting isoleucine improved survival outcomes of male mice. These findings suggest that each BCAA has distinct and sex‐specific effects, with significant uncoupling between metabolic, cognitive, and neuropathological outcomes. Taken together, these results provide new insights into the distinct effects of each BCAA in delaying the progression of AD, will guide future studies into the role of diet in AD development and progression, and highlight BCAA restriction as a promising nutritional approach in slowing or preventing the progression of this devastating disease.

## 4 Materials and Methods

### 4.1 Animals

All procedures were performed in accordance with institutional guidelines and were approved by the Institutional Animal Care and Use Committee (IACUC) of the William S. Middleton Memorial Veterans Hospital and the University of Wisconsin‐Madison IACUC (Madison, WI, USA). Male and female homozygous 3xTg‐AD mice were obtained from The Jackson Laboratory (Bar Harbor, ME, USA) and were bred and maintained at the vivarium with food and water available ad libitum. Prior to the start of the experiments, at 6 months of age, mice were randomly assigned to different groups based on their body weight and diet. Mice were acclimatized on a LabDiet 5001 Rodent Diet for 1 week before randomization. Animals of each sex and strain were randomized at the cage level into groups with comparable starting body weight and body composition prior to assigning them to different diet groups. As each of the diets was color‐coded and cages were labeled based on the diet color, blinding of investigators was not feasible during metabolic and behavioral phenotyping studies. However, histological assessments and image quantification were performed in a blinded manner whenever possible. All mice were maintained at a temperature of approximately 22°C, and health checks were completed on all mice daily.

At the start of the experiment, mice were randomized to four different groups (1) Control (Amino Acid defined diet; Envigo TD.140711), (2) Isoleucine Restricted (IleR; Envigo TD.160734), (3) Leucine Restricted (LeuR, Envigo TD.160736), and (4) Valine Restricted (ValR; Envigo TD.160735) groups. Diet descriptions, compositions, and item numbers are provided in Table S1.

### 4.2 In Vivo Procedures

A glucose tolerance test was performed by fasting the mice overnight and then injecting glucose (1 g kg^−1^) intraperitoneally (i.p.) as previously described [93, 94]. For insulin tolerance, we fasted the mice for 4 h and injected insulin intraperitoneally (0.75 U kg^−1^). Glucose measurements were taken using a Bayer Contour blood glucose meter (Bayer, Leverkusen, Germany) and test strips. Mouse body composition was determined using an EchoMRI Body Composition Analyzer (EchoMRI, Houston, TX, USA). For determining metabolic parameters [O2, CO2, food consumption, respiratory exchange ratio (RER), energy expenditure] and activity tracking, the mice were acclimated to housing in an Oxymax/CLAMS‐HC metabolic chamber system (Columbus Instruments) for ∼24 h, and data from a continuous 24 h period were then recorded and analyzed. Mice were euthanized by cervical dislocation after a 3 hr fast, and tissues for molecular analysis were flash‐frozen in liquid nitrogen or fixed and prepared as described in the methods below.

### 4.3 Behavioral Assays

All mice underwent behavioral phenotyping at 12 months of age. The Novel object recognition test (NOR) was performed in an open field where the movements of the mouse were recorded via a camera that is mounted above the field. Before each test, mice were acclimatized in the behavioral room for 30 min and were given a 5 min habituation trial with no objects in the field. This was followed by test phases that consisted of two trials that were 24 h apart: Short‐term memory test (STM and Long‐term memory test (LTM). In the first trial, the mice were allowed to explore two identical objects placed diagonally on opposite corners of the field for 5 min. Following an hour after the acquisition phase, STM was performed, and 24 h later, LTM was done by replacing one of the identical objects with a novel object. The results were quantified using a discrimination index (DI), representing the duration of exploration for the novel object compared to the old object.

For Barnes maze, the test involves 3 phases: habituation, acquisition training, and the memory test. During habituation, mice were placed in the arena and allowed to freely explore the escape hole, escape box, and the adjacent area for 2 min. Following that, during acquisition training, the mice were given 180 s to find the escape hole, and if they failed to enter the escape box within that time, they were led to the escape hole. After 4 days of training, on the 5th day (STM) and 12th day (LTM), the mice were given 90 s memory probe trials. The latency to enter the escape hole, distance traveled, and average speed were analyzed using Ethovision XT (Noldus).

### 4.4 Immunoblotting

Tissue samples from the left hemisphere of the brain were lysed in cold RIPA buffer supplemented with phosphatase inhibitor and protease inhibitor cocktail tablets (Thermo Fisher Scientific, Waltham, MA, USA) using a FastPrep 24 (M.P. Biomedicals, Santa Ana, CA, USA) with bead‐beating tubes (16466–042) from (VWR, Radnor, PA, USA) and zirconium ceramic oxide bulk beads (15340159) from (Thermo Fisher Scientific, Waltham, MA, USA). Protein lysates were then centrifuged at 13,300 rpm for 10 min, and the supernatant was collected. Protein concentration was determined by Bradford (Pierce Biotechnology, Waltham, MA, USA). 20 µg protein was separated by SDS–PAGE (sodium dodecyl sulfate–polyacrylamide gel electrophoresis) on 8%, 10%, or 16% resolving gels (ThermoFisher Scientific, Waltham, MA, USA) and transferred to PVDF membrane (EMD Millipore, Burlington, MA, USA). The phosphorylation status of mTORC1 substrates p‐S240/S244 S6 and 4E‐BP1 T37/S46 was assessed in the brain along with autophagy markers, including autophagy proteins ATG5, ATG7, and ATG16L1, as well as autophagosome formation proteins Beclin and light chain 3A/B (LC3A/B), and the autophagy receptor p62 (sequestosome 1, SQSTM1). Tau pathology was assessed by Western blotting with anti‐tau antibody. Antibody vendors, catalog numbers, and the dilution used are provided in Table S9. Imaging was performed using a Bio‐Rad Chemidoc MP imaging station (Bio‐Rad, Hercules, CA, USA). Quantification was performed by densitometry using NIH ImageJ software.

### 4.5 Histology for AD Neuropathology Markers

Mice were euthanized by cervical dislocation after 3 h fast, and the right hemisphere was fixed in formalin for histology, whereas the left hemisphere was snap‐frozen for biochemical analysis. For amyloid plaque staining, briefly, brain sections were deparaffinized and rehydrated according to standard protocol. For epitope retrieval, mounted slides were pretreated in 70% formic acid at room temperature for 10 min. Tissue sections were subsequently blocked with normal goat serum (NGS) at room temperature for 1 hr, then incubated with monoclonal antibodies 6E10 (1:100) at 4°C overnight. Aβ immunostained profiles were visualized using diaminobenzidine chromagen. For p‐Tau staining and glial activation, brains were analyzed with p‐Tau Thr231, anti‐GFAP (astrocytic marker), and anti‐Iba1 (microglial marker) antibodies, respectively. The following primary antibodies were used: phospho‐Tau (Thr231) monoclonal antibody (AT180) (Thermo Fisher Scientific; # MN1040, 1:100) anti‐GFAP (Thermo Fisher; # PIMA512023; 1:1,000), anti‐IBA1 (Abcam; #ab178847; 1:1,000). Sections were imaged using an EVOS microscope (Thermo Fisher Scientific Inc., Waltham, MA, USA) at a magnification of 4X, 10X, and 40X. Image‐J was used for quantification by converting images into binary images via an intensity threshold, and the positive area was quantified.

### 4.6 Transcriptomic Analysis

RNA was extracted from the left hemisphere of the brain using trizol followed by Purelink RNA mini kit (cat#12183018A; Thermo Fisher Scientific, Waltham, MA). The concentration and purity of RNA were determined using a NanoDrop 2000c spectrophotometer (Thermo Fisher Scientific, Waltham, MA), and RNA was diluted to 100–400 ng/µl for sequencing. The RNA was then submitted to the University of Wisconsin‐Madison Biotechnology Center Gene Expression Center & DNA Sequencing Facility, and RNA quality was assayed using an Agilent RNA NanoChip. RNA libraries were prepared using the TruSeq Stranded Total RNA Sample Preparation protocol (Illumina, San Diego, CA) with 250ng of mRNA, and cleanup was done using RNA Clean beads (lot #17225200). Reads were aligned to the mouse (Mus musculus) with genome‐build GRCm38.p5 of accession NCBI:GCA_000001635.7, and expected counts were generated with Ensembl gene IDs74.

Analysis of significantly differentially expressed genes (DEGs) was completed in R version 3.4.3 [95] using *edgeR* [96] and *limma* [97]. Gene names were converted to gene symbol and Entrez ID formats using the mygene package. Male and female mice were analyzed separately, and clear outliers were removed following PCA analysis of the raw data. Genes with too many missing values were removed; if genes were present in less than one diet/age group, they were removed. To reduce the impact of external factors not of biological interest that may affect expression, the data were normalized to ensure the expression distributions of each sample are within a similar range. We normalized using the trimmed mean of M‐values (TMM), which scales to library size. Heteroscedasticity was accounted for using the voom function, DEGs were identified using an empirical Bayes moderated linear model, and log coefficients and Benjamini‐Hochberg (BH) adjusted *p*‐values were generated for each comparison of interest [98]. Pathway enrichment was conducted using KEGG pathways on significant genes for each BCAA restricted group vs Control (BH Adjusted *p* < 0.05) using the *limma* package.

WGCNA analysis was conducted in R using the WGCNA package [99] as we have previously described on rankz‐transformed gene expression data [100]. Among all sequenced transcripts in male and female mice (18 865), ∼99% and ∼95% were included in 20 and 19 modules, respectively. Gene expression and WGCNA module gene membership are available at https://connect.doit.wisc.edu/dlamming_BCAA_AD/.

### 4.7 Correlation Analysis

The function *corrplot* in R was used to generate heatmaps to illustrate sex‐specific trait–trait, trait‐WGCNA module eigengene (ME), and ME‐ME relationships. All traits were first rankz‐transformed to yield a normal distribution. Only those relationships satisfying a significance level of *p*‐value < 0.05 are shown. Red indicates a positive Pearson correlation coefficient, blue a negative Pearson correlation coefficient. Correlation data and heatmaps are available at https://connect.doit.wisc.edu/dlamming_BCAA_AD/.

### 4.8 Cytoscape

ME‐trait correlations were used to construct a correlation network in Cytoscape (v3.10.4) where the nodes are co‐expression gene modules denoted by their ME.colorname or physiological traits. Edges connecting nodes represent the correlation between a Module Eigengene (ME) and rankz‐transformed values for all traits. Color indicates direction of correlation (red = positive, blue = negative), thickness is controlled by |correlation| (range, 0.5 to 1.0), and transparency is set by the ‐log_10_ *p*‐value (range, 1 to 5). The relative position of the nodes and curved edges was determined by the yFiles Radial Layout option in Cytoscape.

### 4.9 Metabolomics

For aqueous metabolites extraction, serum (10 µL) was mixed with 300 µl of extraction solvent (40:40:20 methanol:acetonitrile:water, v:v:v) at −20 °C, vortexed, and immediately centrifuged at 16,000g for 20 min at 4 °C. The supernatant (70 µl) was collected for LC–MS analysis. Frozen tissue samples were ground at liquid nitrogen temperature with a CryoMill (Retsch). The resulting tissue powder (approximately 20 mg) was weighed and then mixed with −20 °C extraction solvent containing 0.5% formic acid (40 µl per mg tissue), vortexed, and neutralized with 15% NH4HCO3 (3.5 µl per mg tissue). Following vortexing and centrifugation at 16,000g for 20 min at 4 °C, the supernatant (70 µl) was loaded into LC–MS vials. Metabolites were analyzed by a quadrupole–orbitrap mass spectrometer (Q‐Exactive Plus Hybrid Quadrupole–Orbitrap, Thermo Fisher) coupled to hydrophilic interaction chromatography by heated electrospray ionization. LC separation was performed on an Xbridge BEH amide column (2.1 mm × 150 mm, 2.5 µm particle size, 130 Å pore size; Waters) at 25 °C using a gradient of solvent A (5% acetonitrile in water with 20 mM ammonium acetate and 20 mM ammonium hydroxide) and solvent B (100% acetonitrile). The flow rate was 150 µl min−1. The LC gradient was: 0 min, 90% B; 1.2 min, 90% B; 1.8 min, 75% B; 3 min, 75% B; 4.2 min, 75% B; 4.8 min, 70% B; 5.4 min, 70% B; 6 min, 50% B; 7.2 min, 50% B; 7.8 min, 25% B; 8.4 min, 20% B; 9 min, 20% B; 9.6 min, 0% B; 12.3 min, 0% B; 12.6 min, 90% B; and 15 min, 90% B. Autosampler temperature was set at 4 °C and the injection volume of the sample was 3 µl. MS analysis was acquired in negative and positive ion modes with Full MS scan mode from m/z 70 to 830 and 140,000 resolution with the following operational parameters: AGC target, 3 × 106; maximum IT, 500 ms; sheath gas flow rate, 40; aux gas flow rate, 10; sweep gas flow rate, 2; spray voltage, +3.8 kV and −3.5 kV; spray current, 33 µA; capillary temperature, 300 °C; s‐lens RF level, 50; aux gas heater temperature, 360 °C. For full MS, the parameters were: resolution, 70,000; AGC target, 1 × 106; maximum IT, 200 ms. Data was analyzed using the EI‐MAVEN software.

### 4.10 Statistical Analysis

All statistical analyses were conducted using Prism, version 9.0.2 (GraphPad Software Inc., San Diego, CA, USA). Tests involving multiple factors were analyzed by either a two‐way analysis of variance (ANOVA) with diet and time or sex as variables or by one‐way ANOVA, followed by a Dunnett's post‐hoc test as specified in the figure legends. Alpha was set at 5% (*p* < .05 considered to be significant). Kaplan–Meir survival analysis of 3xTg mice was performed with log‐rank comparisons stratified by sex and diet. Sample sizes (n) for each experimental group are indicated in the figure legends. Data are presented as the mean ± SEM unless otherwise specified.

## Author Contributions

R.B., M.M.S., F.X., I.M.F., C.L.G., M.F.C., M.E.T., A.T., R.M., C.Y.Y., I.G., S.S., and B.A.K. conducted the experiments. R.B., C.L.G., D.V., S.Y., C.I.O., M.P.K., and D.W.L. analyzed the data. R.B., C.L.G., M.J.R., L.P., D.A.H., M.P.K., A.D.A., C.J., and D.W.L. wrote and edited the manuscript.

## Conflicts of Interest

DWL has received funding from, and is a scientific advisory board member of, Aeovian Pharmaceuticals, which seeks to develop novel, selective mTOR inhibitors for the treatment of various diseases. The remaining authors declare no conflicts of interest.

## Supporting information




**Supporting File 1**: advs74632‐sup‐0001‐SuppMat.pdf.

**Supporting File 2**: advs74632‐sup‐0002‐TablesS1‐S11.zip.

**Supporting File 3**: advs74632‐sup‐0003‐SourceDataBlots.pdf.

## Data Availability

RNA‐sequencing data have been deposited with the Gene Expression Omnibus and are available under accession number GSE299928. The authors declare that source data supporting the findings of this study are available within the paper and its supplementary information and Source Data files. An interactive exploration of all the transcriptomic and phenotypic data generated in this study is available via a web‐based application at: https://connect.doit.wisc.edu/dlamming_BCAA_AD/
