# Supplementary material for: Restriction of Individual Branched‐Chain Amino Acids has Distinct Effects on the Development and Progression of Alzheimer's Disease in 3xTg Mice
Source: Adv Sci (Weinh). 2026 Mar 12;13(30):e15220. doi: 10.1002/advs.202515220 (PMC13248761; doi:10.1002/advs.202515220)
Supplement: Supplementary file 1 — Supporting File 1: advs74632‐sup‐0001‐SuppMat.pdf. [file ADVS-13-e15220-s003.pdf]

Supplementary Figure 1

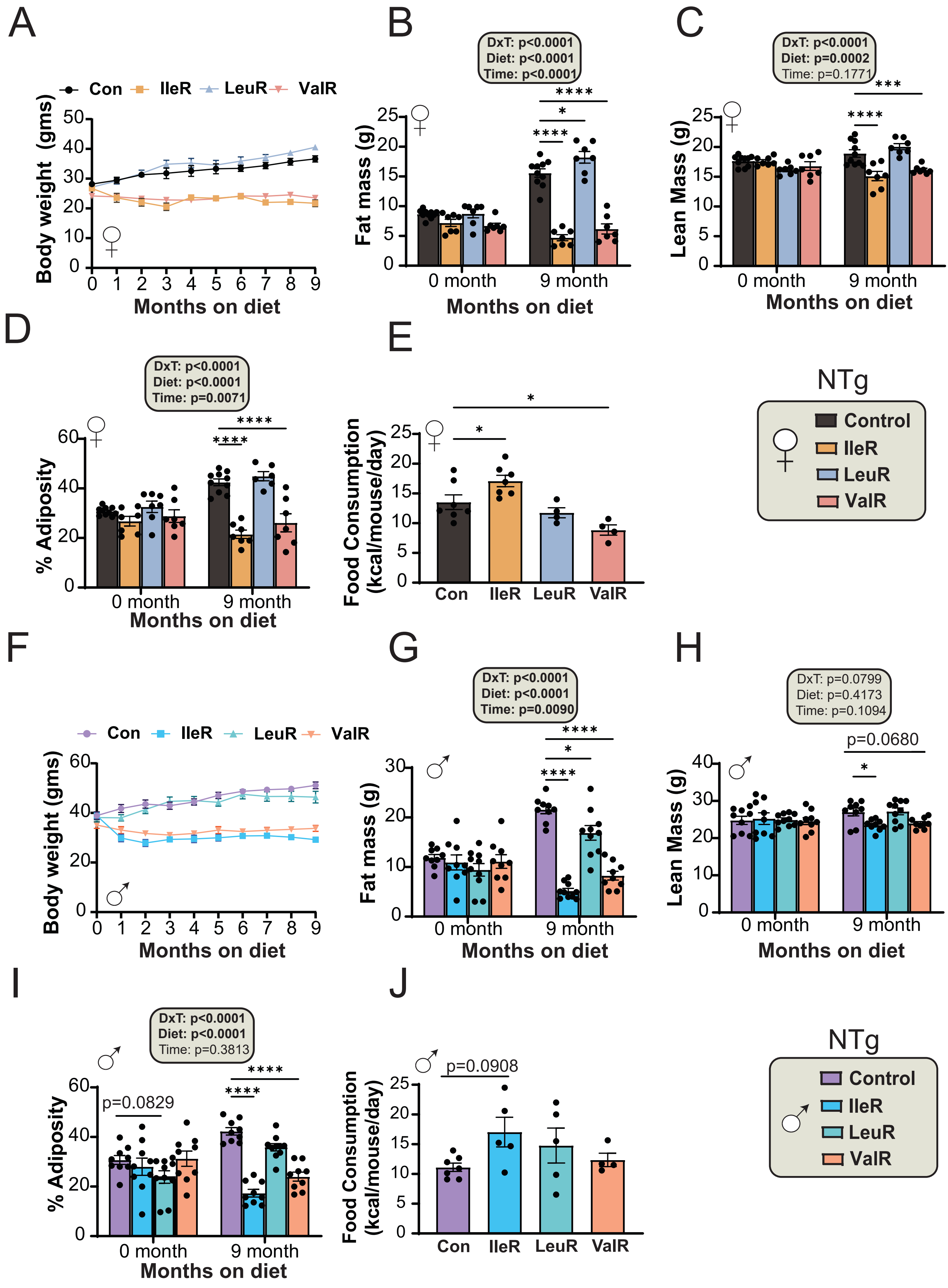

Supplementary Figure Legends

Supplementary Figure 1: Metabolic health outcomes of NTg mice following individual BCAA restriction.

Six-month-old female and male NTg mice were placed on amino acid defined Control (Con) diet or on a diet with a 67% reduction of either isoleucine (IleR), leucine (LeuR), or valine (ValR), and phenotyped over the course of the next 9 months. (A-D) The body weight (A) of female mice was followed over the course of the experiment, fat mass (B) and lean mass (C) was determined at the start and end of the experiment, and the adiposity (D) was calculated. (A-D) n=10 Con, n=7 IleR, n=10 LeuR, and n=9 ValR fed biologically independent NTg female mice. (E) Food consumption of female NTg mice (n=7 Con, n=7 IleR, n=4 LeuR and n=4 ValR fed biologically independent mice). (F-I) The body weight (F) of male mice was followed over the course of the experiment, fat mass (G) and lean mass (H) was determined at the start and end of the experiment, and the adiposity (I) was calculated. (F-I) n=9 Con, n=10 IleR, n=10 LeuR, and n=9 ValR biologically independent NTg male mice. (J) Food consumption of male NTg mice (n=7 Con, n=5 IleR, n=5 LeuR, and n=6 ValR fed biologically independent mice). (B-D, F-I) Statistics for the overall effect of diet and time represent the p value from a two-way analysis of variance (ANOVA); *p<0.05, statistics for the overall effects of diet and time represent the p value from a 2-way ANOVA conducted separately for each time point; *p<0.05, **p<0.01, ***p<0.001, **** p<0.0001 from a Dunnett's post-test examining the effect of parameters identified as significant in the 2-way ANOVA.. (E, J) *p<0.05, one-way ANOVA, followed by a Dunnett's post-test. Data represented as mean ± SEM.

Supplementary Figure 2

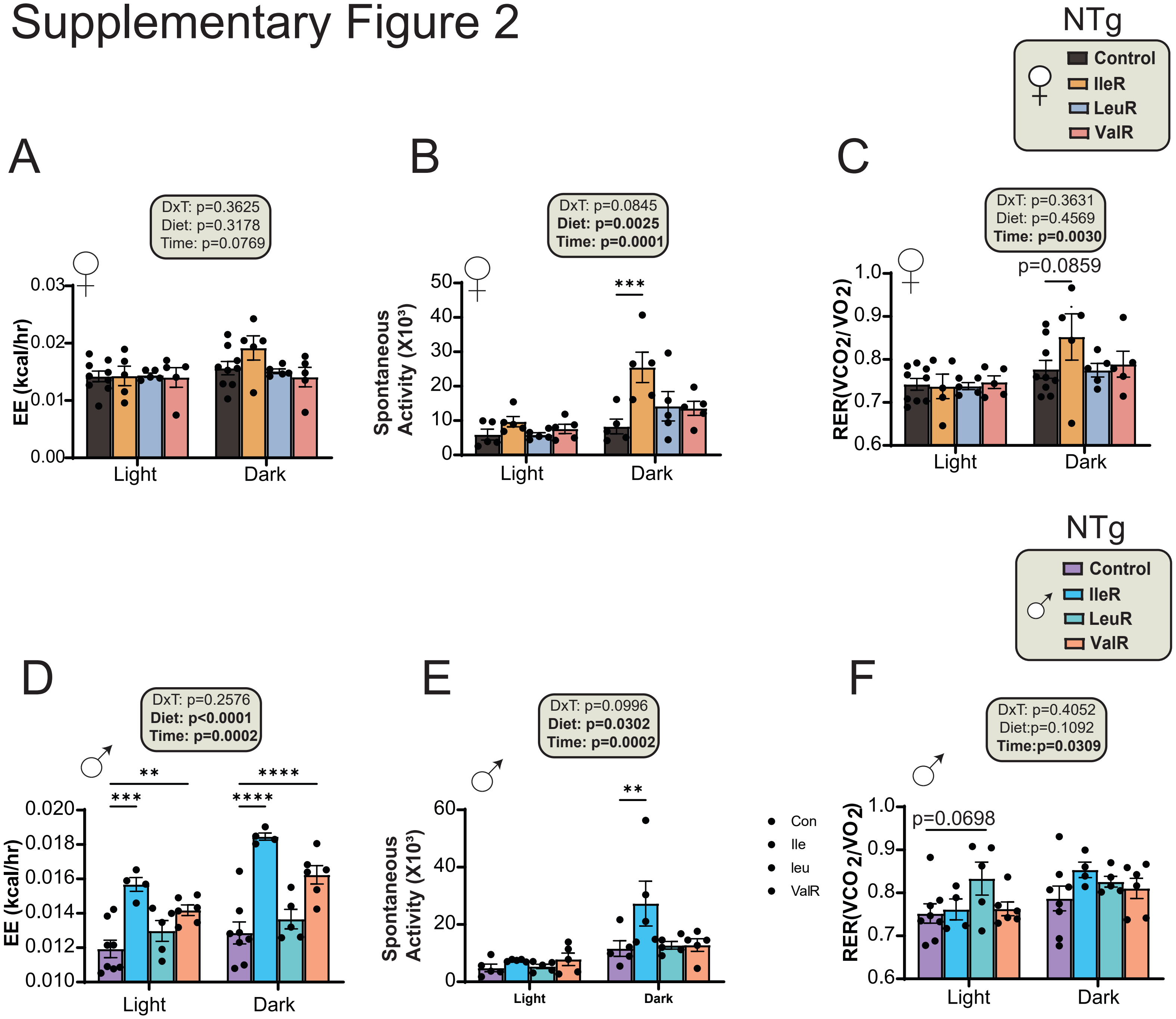

Supplementary Figure 2: Individual BCAA restriction alters energy expenditure and activity in NTg mice in a sex-specific manner.

(A-F) Metabolic chambers were used to determine energy expenditure, spontaneous activity and fuel source utilization, over 24 hours in six-month-old female (A-C) and male (D-F) NTg mice fed the indicated diets for 3 months. (A, D) Energy expenditure normalized to body weight in females (A) and males (D). (B, E) Spontaneous activity of females (B) and males (E). (C, F) Respiratory exchange ratio (RER) in females (C) and males (F). (A-C) n=9 Con, n=5 IleR, n=5 LeuR and n=5 ValR fed NTg biologically independent mice. (D-F) n=8 Con, n=4 IleR, n=5 LeuR and n=6 ValR fed NTg biologically independent mice. (A-F) Statistics for the overall effect of diet and time represent the p value from a two-way ANOVA; **p<0.01, ***p<0.001, **** p<0.0001 Dunnett's post-test examining the effect of parameters identified as significant in the two-way ANOVA. Data represented as mean ± SEM.

Supplementary Figure 3

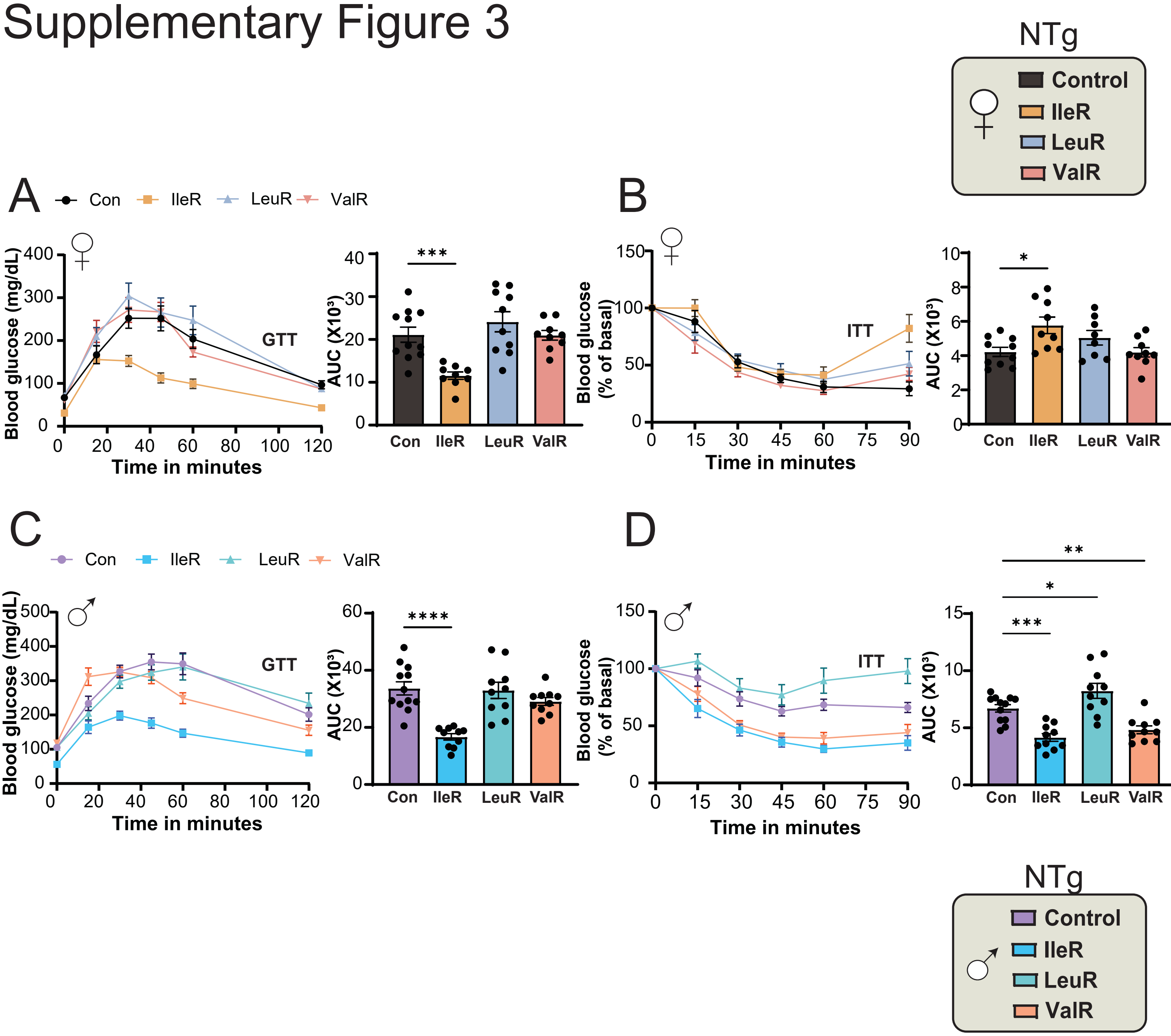

Supplementary Figure 3: Effects of individual BCAA restriction on glucose and insulin tolerance in NTg Mice.

(A-B) Glucose (A) and insulin (B) tolerance tests were performed in female NTg mice fed the indicated diets for 3 months. (A) GTT: n=11 Con, n=9 IleR, n=10 LeuR and n=9 ValR NTg biologically independent mice per group. (B) ITT: n=10 Con, n=9 IleR, n=8 LeuR and n=9 ValR NTg biologically independent mice per group. (C-D) Glucose (C) and insulin (D) tolerance tests were performed in male NTg mice fed the indicated diets for 3 months. (A) GTT: n=12 Con, n=10 IleR, n=10 LeuR and n=10 ValR fed NTg biologically independent mice (B) ITT: n=12 Con, n=10 IleR, n=10 LeuR and n=10 ValR fed NTg biologically independent mice. (A-D) Dunnett's post-test examining the effect of parameters identified as significant in the one-way ANOVA. $^{*}p<0.05$, $^{**}p<0.01$, $^{***}p<0.001$, **** $p<0.0001$. Data represented as mean ± SEM. AUC, Area Under the Curve.

Supplementary Figure 4

3xTg

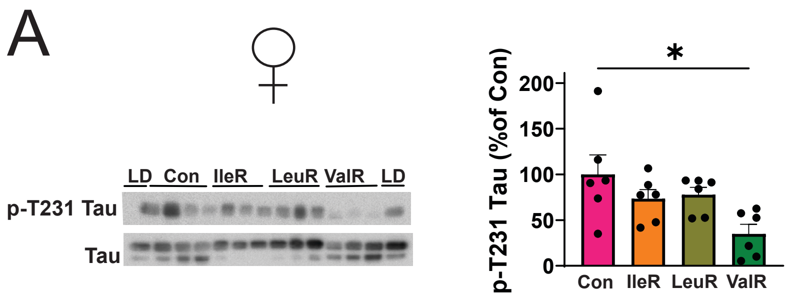

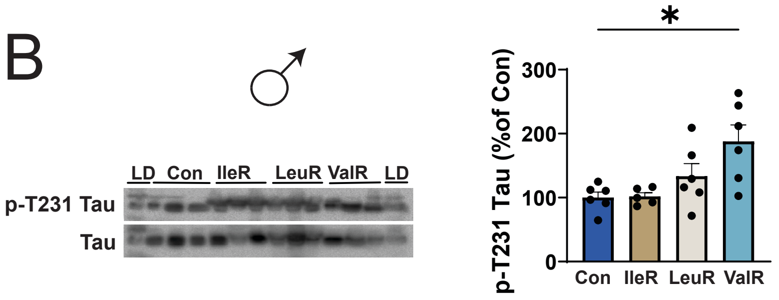

Supplementary Figure 4: Individual BCAA restriction effects on Tau phosphorylation in the whole brain of 3xTg mice

Western blot analysis of phosphorylated T231 Tau in whole brain lysates of (A) female and male (B) 3xTg mice. (A-B) n=6 3xTg biologically independent mice per group. *p<0.05, **p<0.01, ***p<0.001, ****p<0.0001 Dunnett's post-test examining the effect of parameters identified as significant in the one-way ANOVA. Data represented as mean ± SEM.

Supplementary Figure 5

3xTg

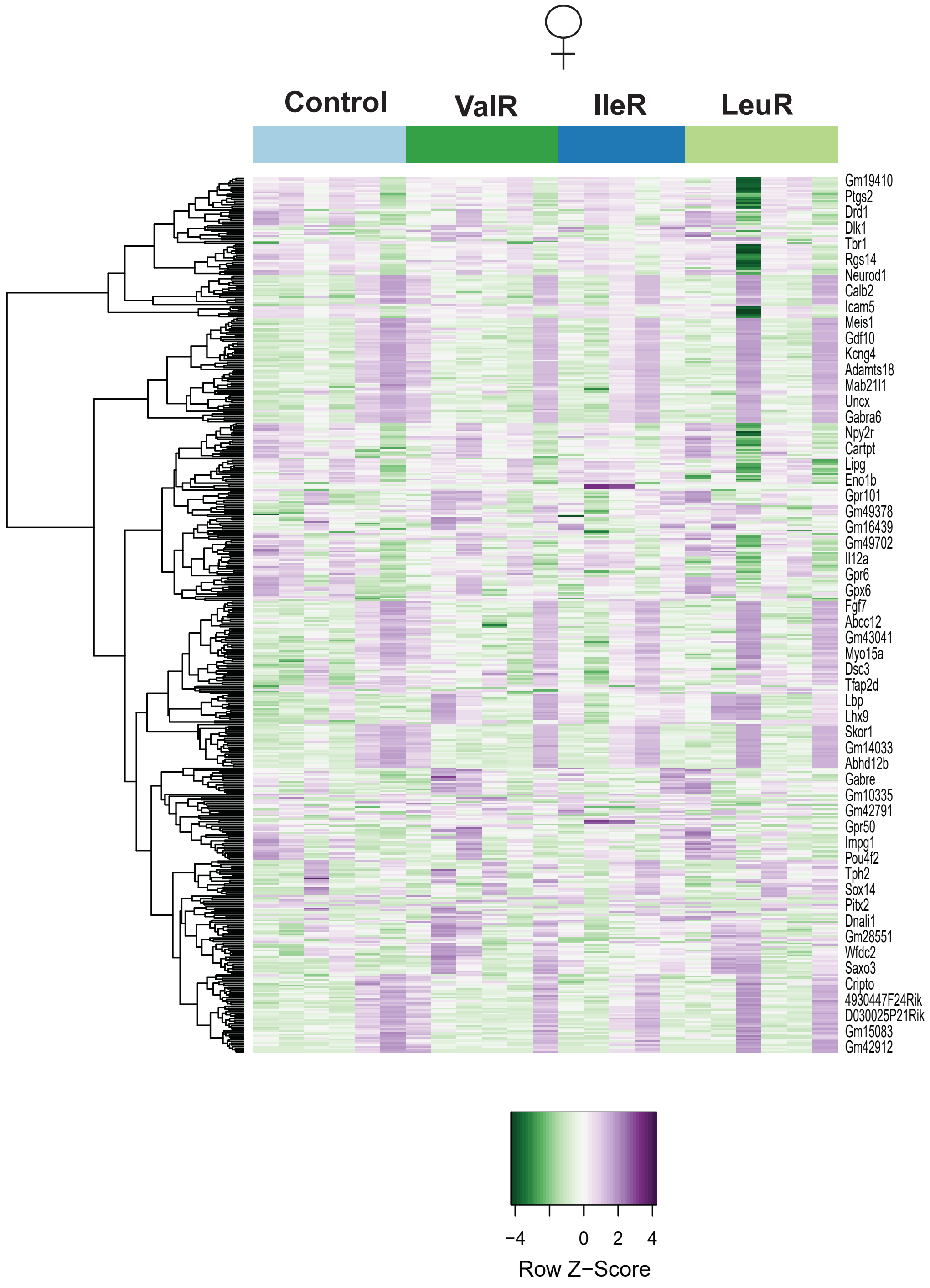

Supplementary Figure 5: Individual BCAA restriction did not impact transcripts in female brain.

Heatmap of the top 50 differentially expressed (DEG) genes in females. n=5-6 animals/group.

Supplementary Figure 6

3xTg

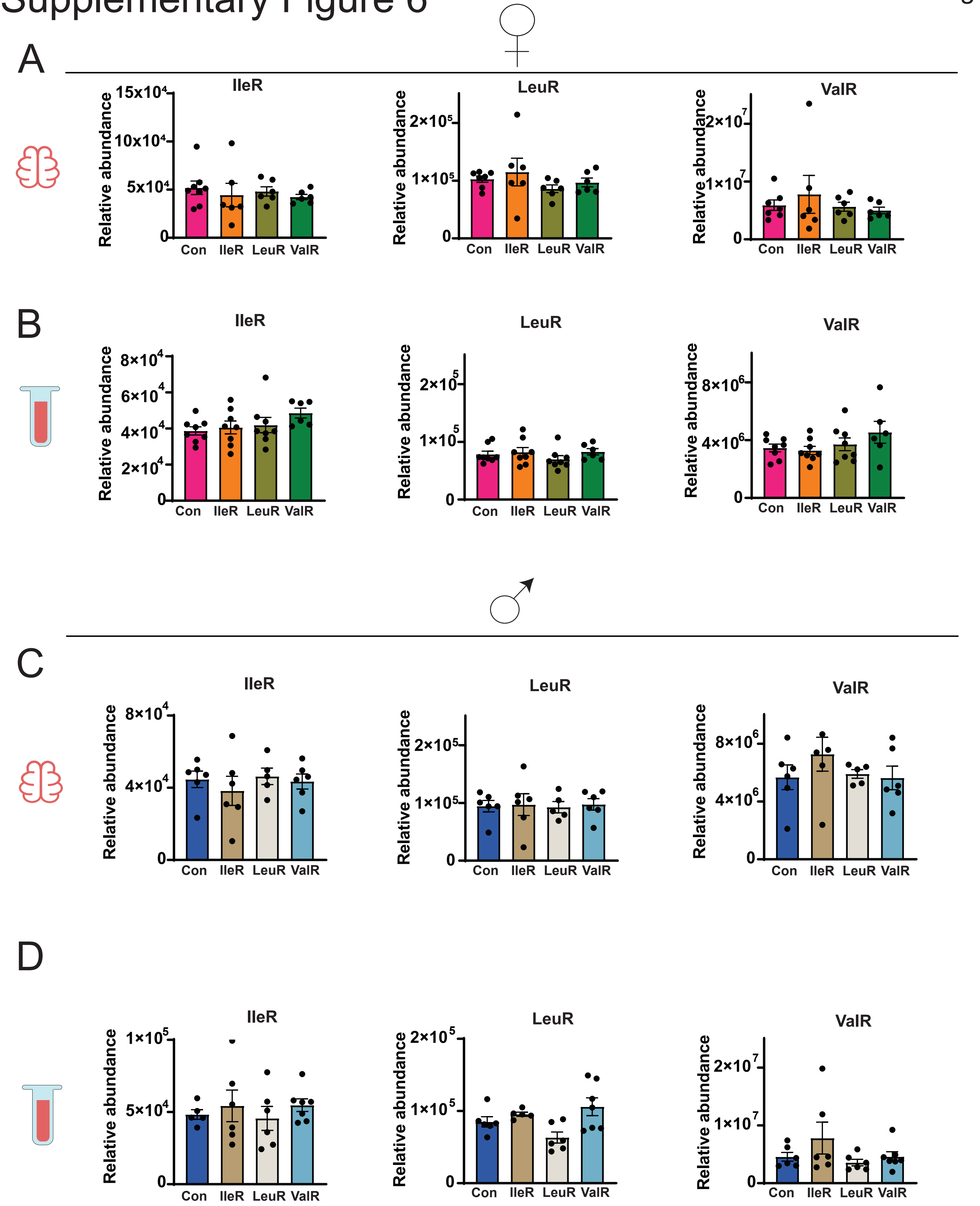

Supplementary Figure 6: Effects of individual BCAA restriction on circulating and brain BCAA levels.

(A-B) Individual BCAA restriction in the brains and serum of female 3xTg mice. (C-D) Individual BCAA restriction in the brains and serum of male 3xTg mice. n=5-8 biologically independent 3xTg mice per group.

Supplementary Figure 7

3xTg

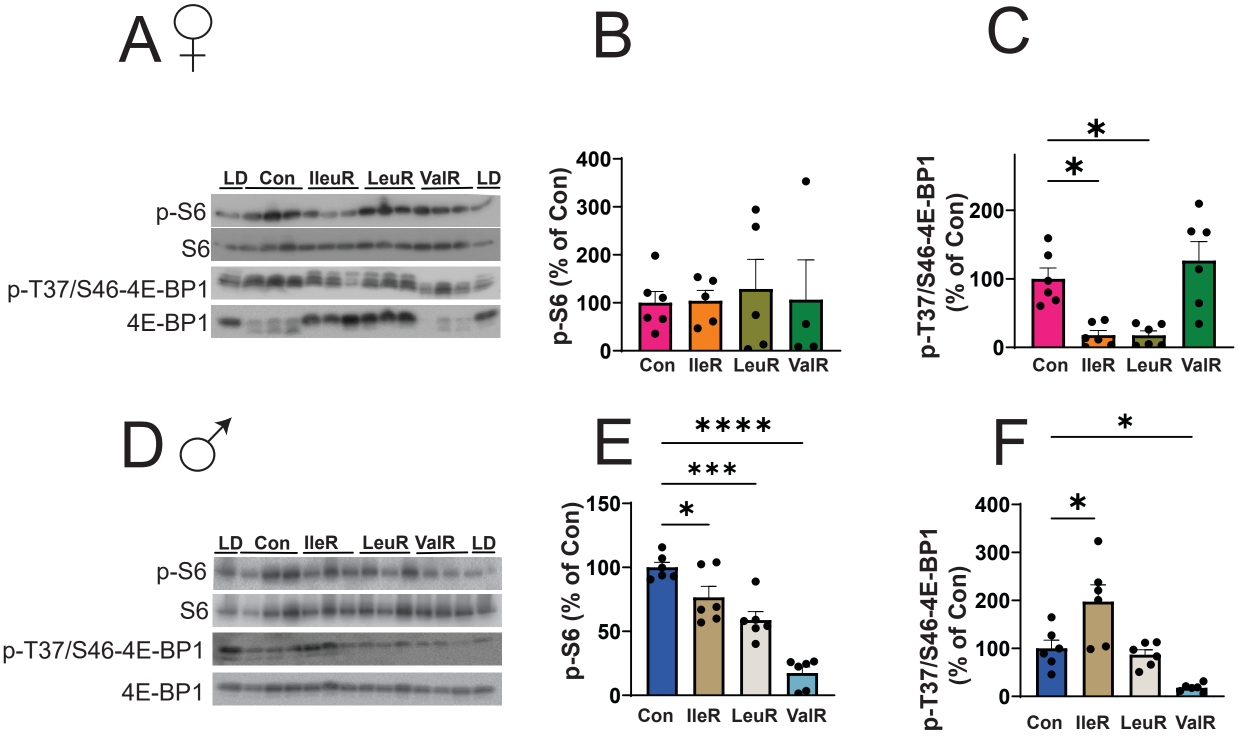

Supplementary Figure 7: mTORC1 signaling in the brain of 3xTg mice following individual BCAA restriction

(A-F) Western blotting of mTORC1 signaling substrates in the whole brain lysates of 3xTg mice. (A, D) Representative immunoblot of p-S240/S244 S6 and T37/S46 4E-BP1 in females (A) and males (D). (B, E) Quantification of the phosphorylation of p-S240/S244 S6 (B) in females and (E) males relative to expression of S6 in (C, F) Quantification of the phosphorylation of T37/S46 4E-BP1 in (C) females and (F) males relative to expression of 4E-BP1. n=5-6 biologically independent 3xTg mice per group. *p<0.05, ***p<0.001, ****p<0.0001 Dunnett's post-test examining the effect of parameters identified as significant in the one-way ANOVA. Data represented as mean ± SEM.

Supplementary Figure 8

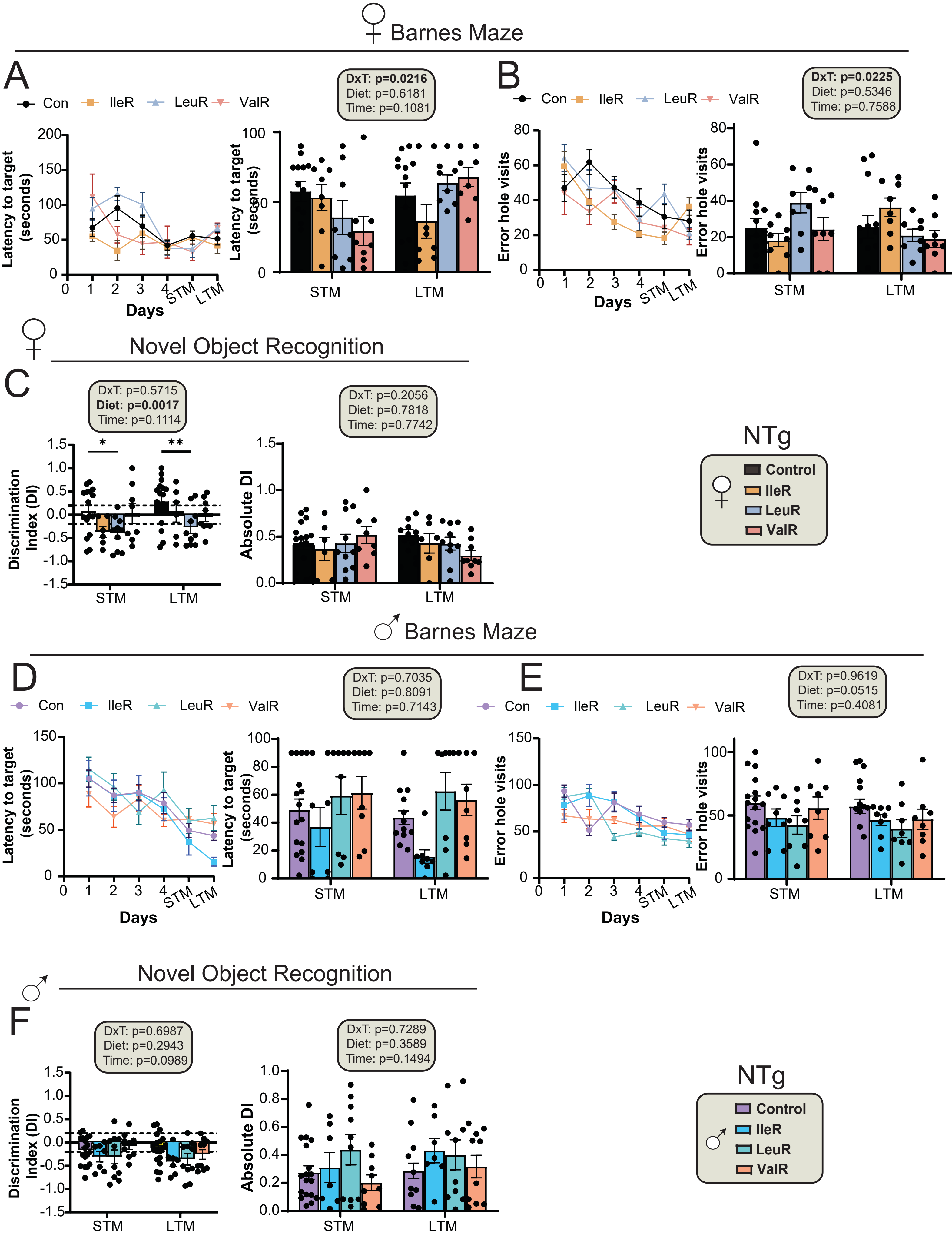

Supplementary Figure 8: Cognitive effects of individual BCAA restriction in NTg Mice.

(A-F) The behavior of female and male NTg mice was examined at 12 months of age after mice were fed the indicated diets for 6 months. (A, D) Latency of target in Barnes Maze acquisition period over the five days of training and in short term memory (STM) and long-term memory (LTM) tests in female (A) and male mice (D). (B, E) The number of error hole visits during Barnes maze training phase in STM and LTM tests by female (B) and male (E) mice. (C, F) The preference for a novel object over a familiar object was assayed in female (C) and male (F) mice via STM and LTM tests. The dashed lines at +0.2 and -0.2 indicate the threshold for discrimination index (DI) values showing the preference for novel or familiar objects. Absolute DI was plotted to show the magnitude of discrimination regardless of the direction of preference. (A-B) n=13 Con, n=8, IleR n=8, LeuR, and n=8 ValR fed biologically independent NTg female mice. (C) n=16 Con, n=6 IleR, n=10 LeuR and n=9 ValR NTg biologically independent NTg female mice per group. (D-E) n=16 Con, n=8 IleR, n=8 LeuR and n=8 ValR fed NTg biologically independent NTg male mice. (F) n=18 Con, n=7 IleR, n=10 LeuR, and n=10 ValR fed biologically independent NTg male mice. (A-F) Statistics for the overall effects of diet, time and the interaction represent the p value from a 2-way ANOVA. *p<0.05, **p<0.01, Dunnett's post-test examining the effect of parameters identified as significant in the two-way ANOVA. Data represented as mean ± SEM.

Supplementary Figure 9

3xTg

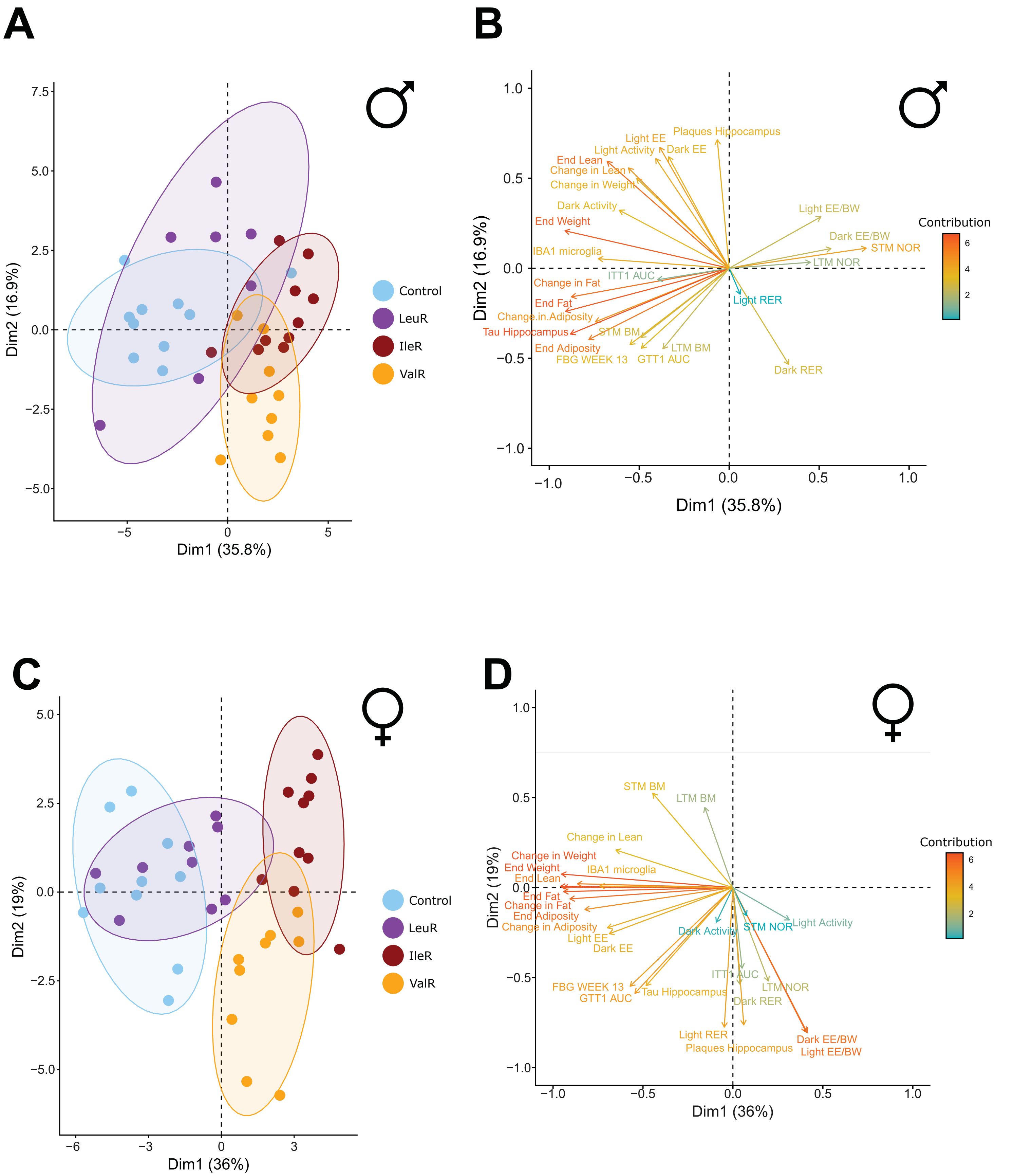

Supplementary Figure 9: Principal component analysis of combined phenotypic traits in both sexes.

Principal component analysis of the phenotypic traits and the variables contributing to the PCA spread in (A-B) in males and (C-D) in females.

Supplementary Table Legends

Supplementary Table 1: Diet composition and calorie content for diets used in this study.

Supplementary Table 2: Differentially expressed gene names, log_2 fold-changes and related p-values from transcriptomic analysis in the brains of male 3xTg mice across diet groups.

Supplementary Table 3: Differentially expressed gene names, log_2 fold-changes and related p-values from transcriptomic analysis in the brains of female 3xTg mice across diet groups.

Supplementary Table 4: KEGG enriched pathways for significant genes identified in 3xTg male mice as shown in **Figure 5C.**

Supplementary Table 5: Pearson correlation analysis between WGCNA module eigengenes and phenotypic traits as shown in **Figure 9C**.

Supplementary Table 6: Cytoscape correlation table input of male 3xTg mice as shown in **Figure 10A.**

Supplementary Table 7: KEGG pathway enrichment analysis of the genes within the Black module in male 3xTg mice as shown in **Figure 10B**.

Supplementary Table 8: KEGG pathway enrichment analysis of the genes within the lightcyan module in male 3xTg mice as shown in Figure 10C.

Supplementary Table 9: Antibodies used for both western blotting and immunohistochemistry.

Supplementary Table 10: Summary of the major phenotypic and molecular outcomes observed in female and male 3xTg mice fed control the indicated diets. Arrows indicate the direction of change relative to Control-fed animals. Up arrow indicates improved/increased; down arrow indicates worsened/decreased; horizontal bi-directional arrow indicates no significant change. Cognitive outcomes are based on Barnes Maze (BM) short-term (STM) and long-term memory (LTM) tests and Novel Object Recognition (NOR). Autophagy and mTORC1 outcomes reflect changes in protein expression measured from whole brain immunoblotting. NS: Non-significant. STM: Short-term memory; LTM: Long-term memory; BM: Barnes Maze; NOR: Novel Object Recognition.

Supplementary Table 11: Summary of genotype dependent vs genotype independent effects of individual BCAA restriction in female and male 3xTg and NTg mice. M, males; F, females; BM, Barnes Maze; NOR, Novel Object Recognition., up arrow indicates significant increase/improvement; down arrow indicates significant decrease/reduction. Bold indicates

effects unique to the 3xTg-AD model, demonstrating that cognitive and neuropathological benefits require AD pathological context, while metabolic effects occur independently of AD pathology.
