# Supplementary material for: Restriction of Individual Branched‐Chain Amino Acids has Distinct Effects on the Development and Progression of Alzheimer's Disease in 3xTg Mice
Source: Adv Sci (Weinh). 2026 Mar 12;13(30):e15220. doi: 10.1002/advs.202515220 (PMC13248761; doi:10.1002/advs.202515220)
Supplement: Supplementary file 2 — Supporting File 2: advs74632‐sup‐0002‐TablesS1‐S11.zip. [file ADVS-13-e15220-s001.zip › Supplementary Table 11 .docx]

**Supplementary Table 11**

**Genotype dependent vs Genotype independent effects of individual BCAA restriction**

| Category | Outcome | BCAA | Genotype |
| --- | --- | --- | --- |
| Genotype Independent Effects |  |  |  |
| Metabolic | Adiposity | IleR, ValR | 3xTg and NTg |
|  | Glucose tolerance | IleR | 3xTg and NTg |
|  | Energy expenditure | ValR | 3xTg and NTg |
| Genotype-Dependent Effects |  |  |  |
| Cognition | Spatial memory (BM) | IleR, LeuR (M), ValR (F) | **3xTg** |
|  | Recognition Index (NOR) | IleR , LeuR, ValR (M only) | **3xTg** |
| Neuropathology |  |  | **3xTg** |
|  | Hippocampal p-Tau | IleR, LeuR, ValR (F only) | **3xTg** |
|  | Amyloid plaques | IleR, LeuR (F only) | **3xTg** |
|  | Microglial activation | IleR, ValR | **3xTg** |
