# Supplementary material for: Restriction of Individual Branched‐Chain Amino Acids has Distinct Effects on the Development and Progression of Alzheimer's Disease in 3xTg Mice
Source: Adv Sci (Weinh). 2026 Mar 12;13(30):e15220. doi: 10.1002/advs.202515220 (PMC13248761; doi:10.1002/advs.202515220)
Supplement: Supplementary file 2 — Supporting File 2: advs74632‐sup‐0002‐TablesS1‐S11.zip. [file ADVS-13-e15220-s001.zip › Supplementary Table 10 .docx]

**Supplementary Table 10**

**Summary of sex-specific metabolic, neuropathological, cognitive, and molecular effects of individual BCAA restriction in 3xTg-AD Mice**

| **Category** | **Outcome** | **Females-IleR** | **Females-LeuR** | **Females-ValR** | **Males -IleR** | **Males-LeuR** | **Males ValR** |
| --- | --- | --- | --- | --- | --- | --- | --- |
| **Metabolic** | Body weight |  |  |  |  |  |  |
|  | Adiposity |  |  |  |  |  |  |
|  | Food intake |  |  |  |  |  |  |
|  | Energy expenditure | (NS) |  |  |  |  |  |
|  | Glucose tolerance | Improved |  |  |  |  |  |
|  | Insulin sensitivity |  |  | Worsened |  |  |  |
| **Neuropathology (Hippocampus)** | Aβ plaques |  |  | (x2) |  |  |  |
|  | p-Tau (Thr231) |  |  |  |  |  |  |
|  | Microglial activation |  |  |  |  |  |  |
| **Autophagy** | ATG5/7/16 |  |  |  |  |  |  |
|  | Beclin |  |  |  |  |  |  |
|  | LC3A/B |  |  |  |  |  |  |
|  | p-62 |  |  |  |  |  |  |
| **mTORC1** | p-S6 |  |  |  |  |  |  |
|  | p-4E-BP1 |  |  |  |  |  |  |
| **Cognition** | STM-BM |  |  |  |  |  |  |
|  | LTM-BM |  |  |  |  |  |  |
|  | STM-NOR |  |  |  |  |  |  |
|  | LTM-NOR |  |  |  |  |  |  |
| **Survival** | Lifespan |  |  |  |  |  |  |

Improved /Increased

Worsened/Decreased

No significant change

STM: Short term memory Test. LTM: Long term memory test, BM: Barnes maze, NOR: Novel Object Recognition test
