# Supplementary figures and images for: Restriction of Individual Branched‐Chain Amino Acids has Distinct Effects on the Development and Progression of Alzheimer's Disease in 3xTg Mice
Source: Adv Sci (Weinh). 2026 Mar 12;13(30):e15220. doi: 10.1002/advs.202515220 (PMC13248761; doi:10.1002/advs.202515220)

Set 1 Females

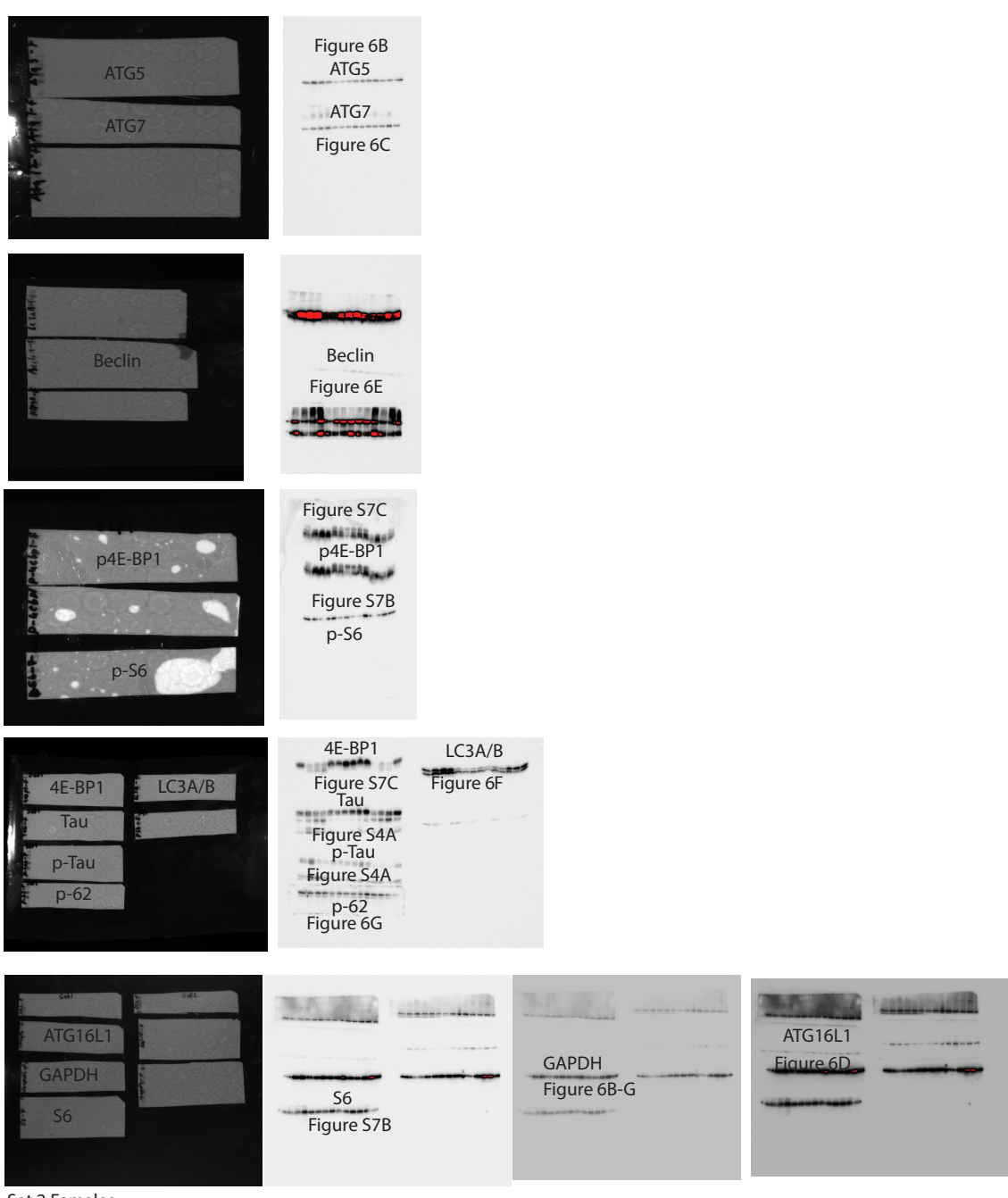

Set 2 Females

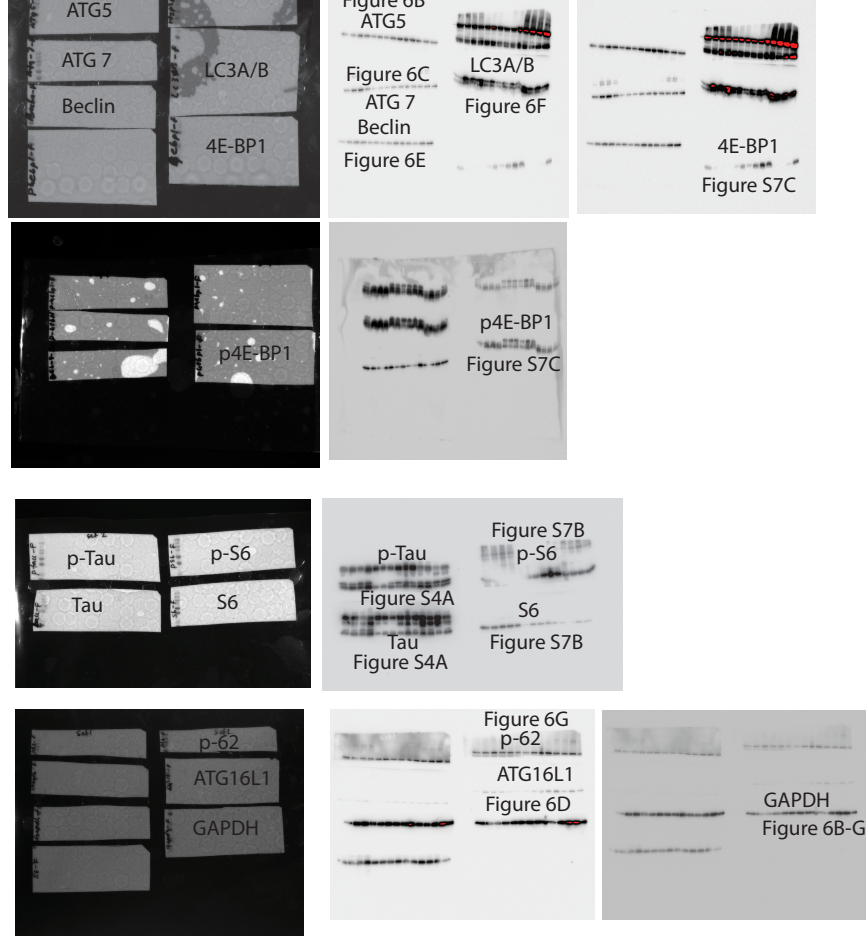

Set 1- Males

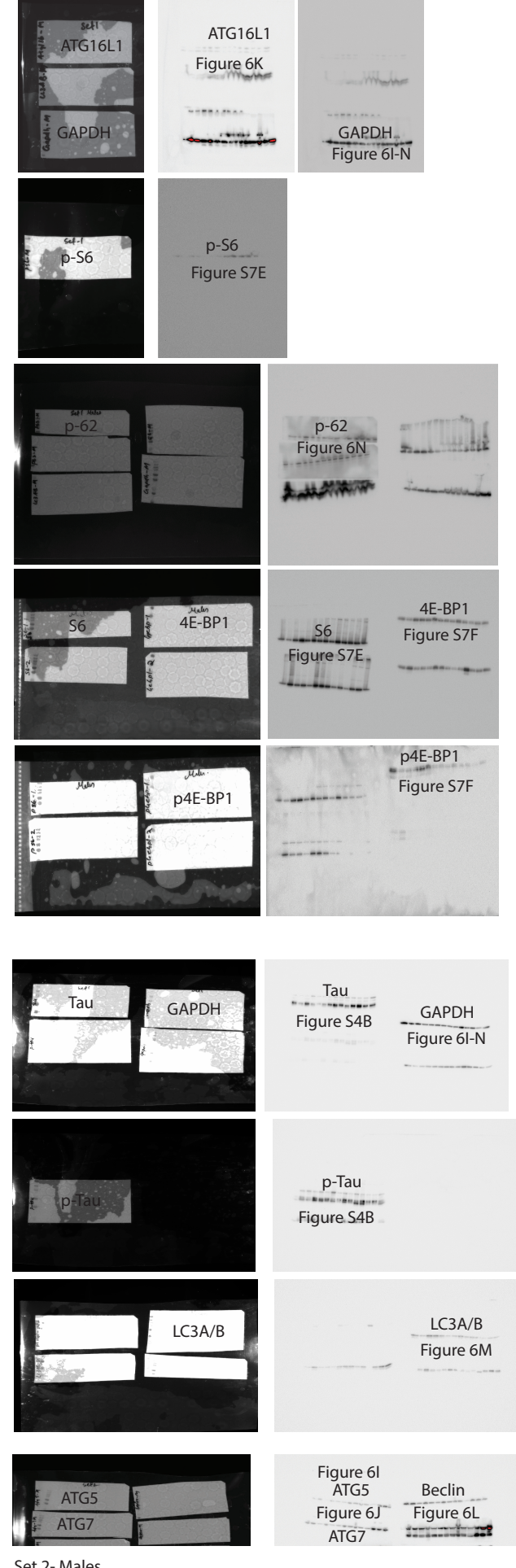

Set 2- Males

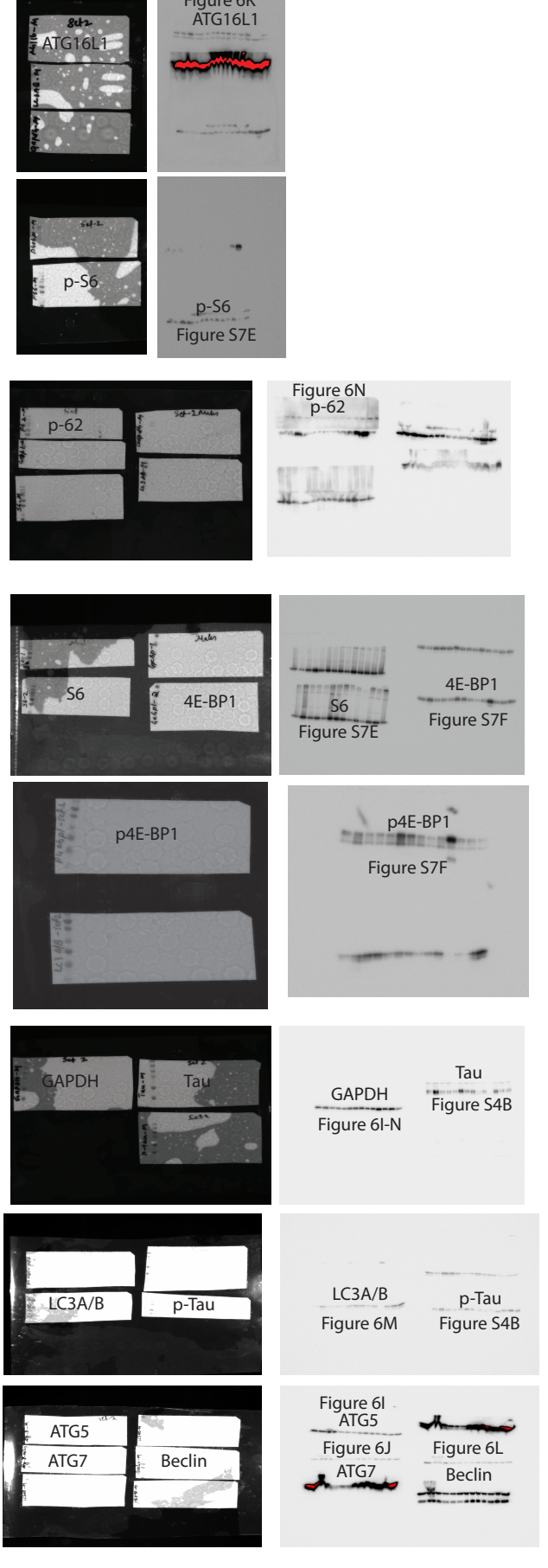

Supplement: Supplementary file 3 — Supporting File 3: advs74632‐sup‐0003‐SourceDataBlots.pdf. [file ADVS-13-e15220-s002.pdf]
